# ClusterMap for multi-scale clustering analysis of spatial gene expression

**DOI:** 10.1038/s41467-021-26044-x

**Published:** 2021-10-08

**Authors:** Yichun He, Xin Tang, Jiahao Huang, Jingyi Ren, Haowen Zhou, Kevin Chen, Albert Liu, Hailing Shi, Zuwan Lin, Qiang Li, Abhishek Aditham, Johain Ounadjela, Emanuelle I. Grody, Jian Shu, Jia Liu, Xiao Wang

**Affiliations:** 1 John A. Paulson School of Engineering and Applied Sciences, Harvard University, Cambridge, MA USA; 2 Broad Institute of MIT and Harvard, Cambridge, MA USA; 3 Department of Chemistry, Massachusetts Institute of Technology, Cambridge, MA USA; 4 Department of Chemistry and Chemical Biology, Harvard University, Cambridge, MA USA; 5 Department of Biological Engineering, Massachusetts Institute of Technology, Cambridge, MA USA; 6 Whitehead Institute for Biomedical Research, Cambridge, MA USA; 7 Cutaneous Biology Research Center, Massachusetts General Hospital, Harvard Medical School, Boston, MA USA

**Keywords:** Data processing, Image processing, Single-cell imaging

## Abstract

Quantifying RNAs in their spatial context is crucial to understanding gene expression and regulation in complex tissues. In situ transcriptomic methods generate spatially resolved RNA profiles in intact tissues. However, there is a lack of a unified computational framework for integrative analysis of in situ transcriptomic data. Here, we introduce an unsupervised and annotation-free framework, termed ClusterMap, which incorporates the physical location and gene identity of RNAs, formulates the task as a point pattern analysis problem, and identifies biologically meaningful structures by density peak clustering (DPC). Specifically, ClusterMap precisely clusters RNAs into subcellular structures, cell bodies, and tissue regions in both two- and three-dimensional space, and performs consistently on diverse tissue types, including mouse brain, placenta, gut, and human cardiac organoids. We demonstrate ClusterMap to be broadly applicable to various in situ transcriptomic measurements to uncover gene expression patterns, cell niche, and tissue organization principles from images with high-dimensional transcriptomic profiles.

## Abstract (web summary)

In situ transcriptomics maps RNA expression patterns across intact tissues taking our understanding of gene expression to a new level. Here, the authors present a computational method that uncovers gene expression, cell niche, and tissue region patterns from 2D and 3D spatial transcriptomics.

## Introduction

Tissue functions arise from the orchestrated interactions of multiple cell types, which are shaped by differential gene expression in three-dimensional (3D) space. To chart the spatial heterogeneity of gene expression in cells and tissues, a myriad of image-based in situ transcriptomics methods (e.g., STARmap, FISSEQ, ISS, MERFISH, seqFISH, osmFISH, etc.) have been developed^1–8^, providing an atlas of subcellular RNA localization in intact tissues. However, it is challenging to directly extract low-dimensional representations of biological patterns from high-dimensional spatial transcriptomic data.

One main challenge is to achieve accurate and automatic cell segmentation that accurately assigns RNAs into individual cells for single-cell analysis. The most common cell segmentation strategy is labeling cell nuclei or cell bodies by fluorescent staining^9–11^ (e.g., DAPI, Nissl, WGA, etc.) and then segmenting the continuous fluorescent signals by conventional or machine learning (ML)-based methods^12^. However, conventional methods, such as distance-transformed watershed^13^, require manual curation to achieve optimal but still unsatisfactory segmentation results. On the other hand, while ML-based methods^14, 15^ can automatically detect the targets (cells) in fluorescent staining, they still require manually annotated datasets for model training and have poor generalization ability to other datasets.

**Fig. 1:**
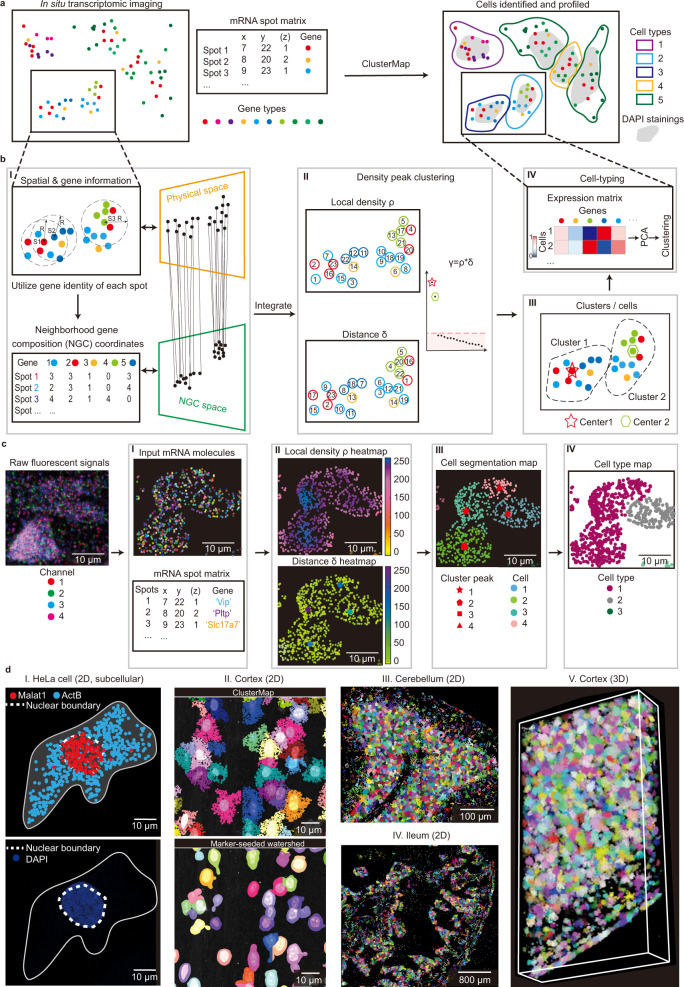
ClusterMap: multi-scale spatial clustering analysis of in situ transcriptomic data from subcellular to tissue scales. **a** Overview of ClusterMap method. The input is a matrix that contains both spatial and transcript information of mRNA molecules sequenced by in situ transcriptomic methods^1–8^. ClusterMap clusters mRNA spots, identifies cells, and profiles them into different cell types as output. **b** Workflow of ClusterMap method. I, The physical and neighborhood gene composition (NGC) coordinates of mRNA spots are extracted for each spot (e.g., S1, S2, and S3), and projected to physical and NGC spaces respectively, which are then computationally integrated. II, Density peak clustering (DPC) algorithm^18^ is used to cluster mRNA in the P-NGC space. III, Each spot is assigned to one cluster, representing one cell. IV, Cell types are identified by the gene expression profiles in each cell. **c** Representative ClusterMap analysis on STARmap mouse V1 1020-gene dataset^6^ corresponds to (I–IV) in **b**. **d** Representative ClusterMap cell segmentation analysis on different samples. I, HeLa cell in two-dimensional (2D) space. The white dashed lines highlight the nuclear boundary identified by the subcellular mRNA distribution from ClusterMap (upper) and DAPI staining (bottom) from the same cell. II. Comparison of ClusterMap (upper) and marker-seeded watershed (bottom) segmentation in mouse visual cortex cells. III, Mouse cerebellum in 2D, 4050 cells. IV, Mouse ileum in 2D, 5550 cells. V, Mouse visual cortex in 3D space, 2251 cells. Width: 309 µm, height: 582 µm, depth: 100 µm.

In order to address these challenges, a fundamentally different approach that bypasses auxiliary cell staining, hyperparameter tuning, and manual labeling is needed. Here, instead of using fluorescent staining, we directly utilized the patterns of spatially resolved RNAs that intrinsically encode high-dimensional gene expression information for subcellular and cellular segmentation, followed by cell-type spatial mapping. To leverage the spatial heterogeneity of RNA-defined cell types, we applied the same strategy to cluster discrete cells into tissue regions. Together, we demonstrated that this computational framework (termed ClusterMap) can identify subcellular structures, cells, and tissue regions (Fig. 1).

## Results

### ClusterMap integrates spatial and gene expression analyses

ClusterMap is based on two key biological phenomena. First, the density of RNA molecules is higher inside cells than outside cells; second, cellular RNAs encoded by different genes are enriched at different subcellular locations, cell types, and tissue regions^16, 17^. Thus, we reasoned that we could identify biologically meaningful patterns and structures directly from in situ transcriptomic data by joint clustering the physical density and gene identity of RNAs. Subsequently, the spatial clusters were interpreted based on the gene identity and spatial scales to represent subcellular localization, cell segmentation, and region identification.

ClusterMap started with pre-processed imaging-based in situ transcriptomic data (Methods), where raw fluorescent images were converted into discrete RNA spots with a physical 3D location and a gene identity (i.e. mRNA spot matrix, Fig. 1a). We reasoned that spatial clusters can be distinguished based on the gene expression in the local neighborhood of each RNA spot. To quantify this, we introduced a high-dimensional vector, termed neighborhood gene composition (NGC), which was computed by considering gene expression profiles in a circular window over each RNA spot (Fig. 1bI, Methods section). ClusterMap is capable of analysis on different spatial resolutions by changing the radius of the window (Supplementary Fig. 2). The size of the window is specifically chosen for the same dataset to match the average size of organelles or cells for subcellular or single-cell analysis, respectively (Methods). The NGC coordinates and physical coordinates of each RNA spot are then computationally integrated into joint physical and NGC (P-NGC) coordinates over each spot.

Next, we aimed to cluster the RNAs in the P-NGC coordinates for downstream segmentation. Out of numerous clustering algorithms, density peak clustering (DPC)^18^, a type of density-based clustering method, was chosen for its versatility in extracting biological features in data and its compatibility with clusters of various shapes and dimensionalities automatically. DPC identifies cluster centers with a higher density than the surrounding regions as well as a relatively large distance from points with higher densities. We applied DPC to compute two variables^18^: local density *ρ* and distance *δ* for each spot in the joint P-NGC space. For each spot, *ρ* value represents the density of its closely surrounded spots, and *δ* value represents the minimal distance to spots with higher *ρ* values. Spots with both high *ρ* and *δ* values are highly likely to be cluster centers. We then ranked the product of these two variables, *γ*, in decreasing order to find genuine clusters with orders of magnitude higher *γ* values (Methods). For example, in Fig. 1b, the two spots with the *γ* values that are orders of magnitude higher than other spots are chosen as cell centers (labeled by a red star and a cyan hexagon, Fig. 1bII). After the two cluster centers (labeled as C1 or C2) have been selected, the remaining spots are assigned to one of the clusters respectively in a descending order of *ρ* value. Each spot is assigned to the same cluster as its nearest previously assigned neighbor^18^, and each cluster of spots represents an individual cell (Fig. 1bIII) for downstream analysis (Fig. 1bIV). Outliers that were falsely assigned among cells can be filtered out using noise detection in DPC^18^. To illustrate this framework, we tested the performance of ClusterMap in five simulated clustering benchmark datasets (Supplementary Fig. 1)^19^ and one representative in situ transcriptomic data collected by STARmap^6^ (Fig. 1c). Compared with previous methods^20^, ClusterMap showed consistent performance in all six datasets even when the spot distributions contained irregular boundary, varying physical density, and heterogeneous shapes and sizes.

Next, we examined and validated the performance of ClusterMap in diverse biological samples at different spatial scales in both 2D and 3D (Fig. 1d). First, based on the assumption that cellular RNAs have a different distribution in the nucleus or cytoplasm^21^, we used ClusterMap to cluster mRNAs within one cell to delineate the nuclear boundary. Here, RNA spots with both highly correlated neighboring composition and close spatial distances were merged into a single signature (Supplementary Fig. 3a and Methods section). Then, a convex hull was constructed from the nucleus spots, denoting the nuclear boundary. The patterns of ClusterMap-constructed nuclear boundaries were highly correlated with DAPI stainings, confirming the power of ClusterMap for segmentation at the subcellular resolution (Fig. 1dI). Second, we compared cell segmentation results by ClusterMap with conventional watershed^13^ segmentation (Methods) on the same mouse cortex cells. Compared to the conventional watershed method, ClusterMap accurately identified cells, more precisely outlined cell boundary and illustrated cell morphology (Fig. 1dII). Last, we extended ClusterMap to diverse types of tissue at different scales in both 2D and 3D, where dense heterogeneous populations of cells with arbitrary shapes exist. Cell identification results for the mouse cerebellum, the ileum, and the cortex are shown in Fig. 1dIII–V.

### Spatial clustering analysis in mouse brain

**Fig. 2:**
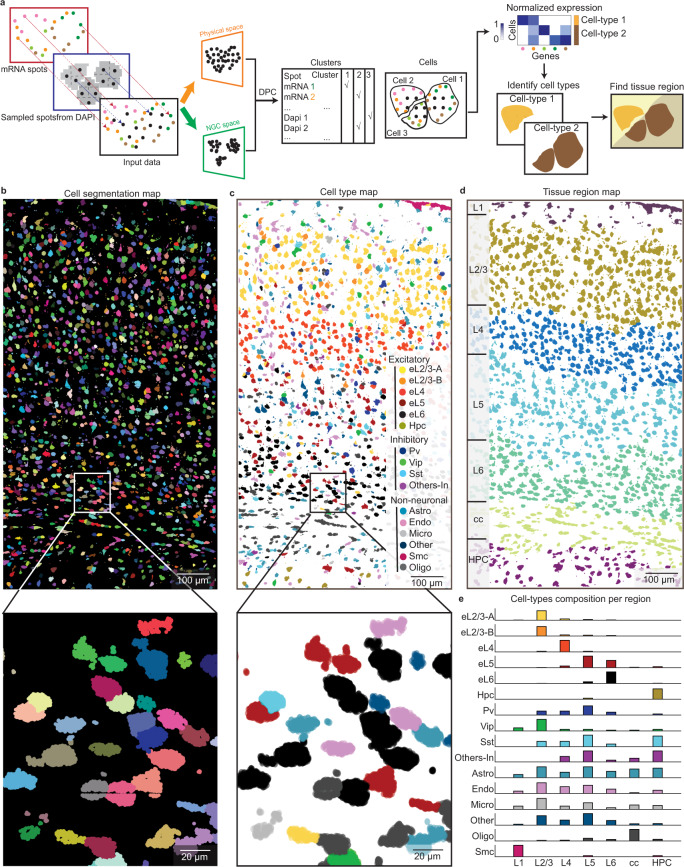
ClusterMap generates cell-type and tissue-region maps in mouse primary cortex (V1). **a** Workflow of ClusterMap method that integrates DAPI signals for spatial clustering. **b**–**d** ClusterMap generates cell (segmentation) map (**b**), cell-type map (**c**), and tissue region map (**d**) of the STARmap mouse V1 1020-gene dataset^6^, which includes 1599 identified cells. **b** mRNA molecules are color-coded by their cell attributes. **c** The cell type names and colorings are from ref. ^6^. Bottom panels in **b**, **c** show the zoomed-in views from the rectangular highlighted regions in upper panels. **d** The tissue regions are segmented and cells in the same layer are shown in the same color. From top to bottom, the tissue region map shows: L1 to L6, the six neocortical layers; cc, corpus callosum; HPC, hippocampus. **e** Bar plots of composition of 16 cell types across 7 layers. Values are normalized in each row. The colors correspond to the cell type legend in **c**.

We first demonstrated ClusterMap on the mouse primary visual cortex from the STARmap mouse primary cortex (V1) 1020-gene dataset^6^ (Supplementary Table 1). When sequenced transcripts were more likely to populate the cytoplasm, sparsely sampled spots based on DAPI signals were combined with RNAs to compensate for the lack of signals in cell nuclei, and they were together processed with ClusterMap procedures (Fig. 2a and Methods section). The results show clear cell segmentation even for strongly crowded mouse V1 cortex cells (Fig. 2b and Supplementary Fig. 3b). Additionally, we evaluated whether ClusterMap-identified cell center coordinates were within corresponding expert-labeled cell regions on eight STARmap mouse V1 datasets to validate its accuracy (Supplementary Fig. 3c). Notably, ClusterMap cell labeling reached accuracy levels of 80–90% compared with manually annotated segmentation labels (Methods section).

In the mouse V1 cortex dataset, ClusterMap identified cell types^22^ that matched both expression signature and tissue localization in the previous report^6^ (Fig. 2c and Supplementary Fig. 4a, b). We further compared the single-cell gene expression profiles from ClusterMap with those from manual annotation, and observed high correlation value and low *p* value between the same cell type (Supplementary Fig. 5a, b and Methods section). Importantly, ClusterMap can consistently identify cell types and spatial localization across different biological replicates in the mouse brain regions (Supplementary Figs. 4c–f and 5c–f).

The next challenge was to apply ClusterMap on the cell-typing map to identify tissue regions. In this case, ClusterMap further clustered cells based on their physical location and cell-type identity, providing similar clustering analyses of physical and high-dimensional cell-type information. ClusterMap computed neighborhood cell-type composition (NCC) coordinates of each cell^23^ and then clustered joint physical and NCC coordinates of cells (Supplementary Fig. 3d and Methods section). As a result, cells with both highly correlated neighboring cell-type composition and close spatial distances are clustered into a single tissue region signature. The results showed that ClusterMap accurately detected cortical layering, which allows for the quantification of cell-type composition of each cortical layer (Fig. 2d, e). The distinct region-specific distribution of excitatory neurons can be observed in the L2/3, L4, L5, and L6 canonical layers, while oligodendrocytes were significantly distributed within the corpus callosum layer. In summary, ClusterMap can effectively, accurately, and automatically conduct cell segmentation, cell typing, and tissue region identification.

### ClusterMap enables spatial clustering and cell niche analyses in mouse placenta

**Fig. 3:**
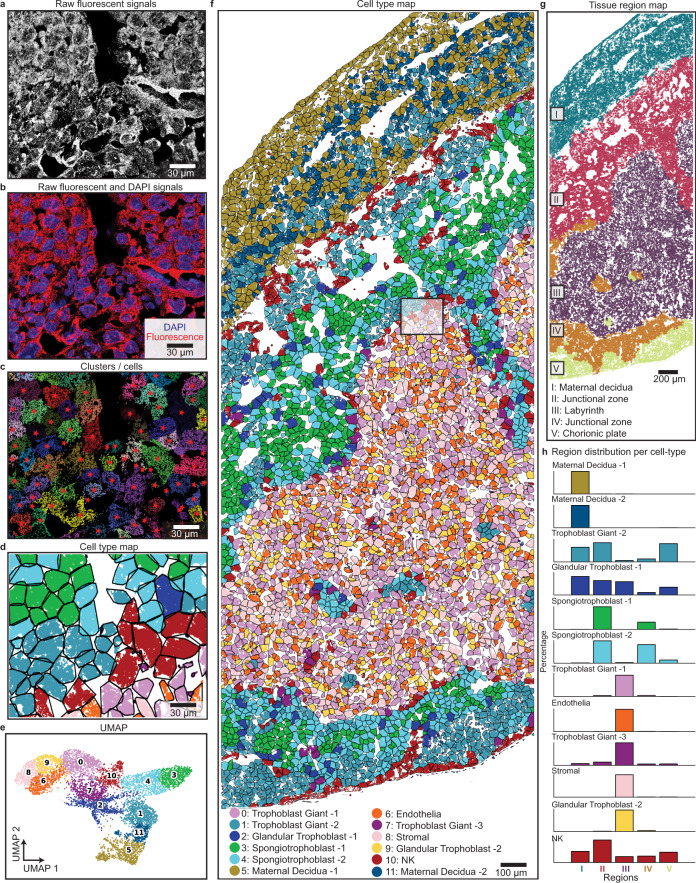
ClusterMap generates cell-type and tissue-region maps in mouse placenta. **a** Raw fluorescent signals for a part in the STARmap mouse placenta 903-gene dataset^6^. Four-channel images in the first sequencing round are overlapped in grayscale to show the mRNA distribution. **b** Composite image by overlapping (**a**) in red and DAPI signals in blue shows the distribution of mRNA relative to cell nuclei. A majority of mRNA molecules distributed outside the cell nucleus, resulting in holes in the cell center. **c**, **d** ClusterMap generates cell segmentation map (**c**) and cell-type map (**d**) of (**a**). Panels **a**–**d** show the zoomed-in view from the highlighted rectangle in **f**, the original dataset. **e** Uniform manifold approximation plot (UMAP) shows clustering of 11 groups across 7224 cells in the original placental dataset. **f** Spatial organization of the cell types in the placental tissue section. The number of cells in each type is as follows: Trophoblast Giant-1 (TG-1), 848; Endothelial (Endo), 578; Stromal (Stro), 418; Trophoblast Giant-2 (TG-2), 833; Maternal Decidua-1 (MD-1), 735; Glandular Trophoblast-1 (GT-1), 717; Spongiotrophoblast-1 (ST-1), 697; Spongiotrophoblast-2 (ST-2), 680; Trophoblast Giant-3 (TG-3), 544; Glandular Trophoblast-2 (GT-2), 410; NK, 404; Maternal Decidua-2 (MD-2), 360. **g** The spatial tissue region map of **f**. **h** Bar plots of composition of 12 cell types across 5 regions. Values are normalized in each row. Cell types in **f**, **h** are color-coded as in **e**.

To further demonstrate the generality of ClusterMap, especially its applicability to tissues with high cell density and variable nuclear/cytosolic distribution of RNAs, we applied ClusterMap to the STARmap mouse placenta 903-gene dataset (Fig. 3a, b and Supplementary Table 1). With ClusterMap analyses described in Fig. 2a, up to 7224 cells were identified (Fig. 3c and Supplementary Fig. 6a) and then clustered into twelve cell types using Louvain clustering^22^, whose marker genes are consistent with cell types defined from single-cell RNA-sequencing (scRNA-seq)^24^ (Fig. 3d–f and Supplementary Fig. 6b–d). ClusterMap identified five tissue regions based on the cell-type map (Fig. 3g), which corresponded to the histological section of a mouse placenta in late gestation (H&E staining)^25^. Further analysis showed that Regions II and IV consisted of similar cell-type compositions, while region I consisted of most maternal decidua (MD) cells (Fig. 3h).

**Fig. 4:**
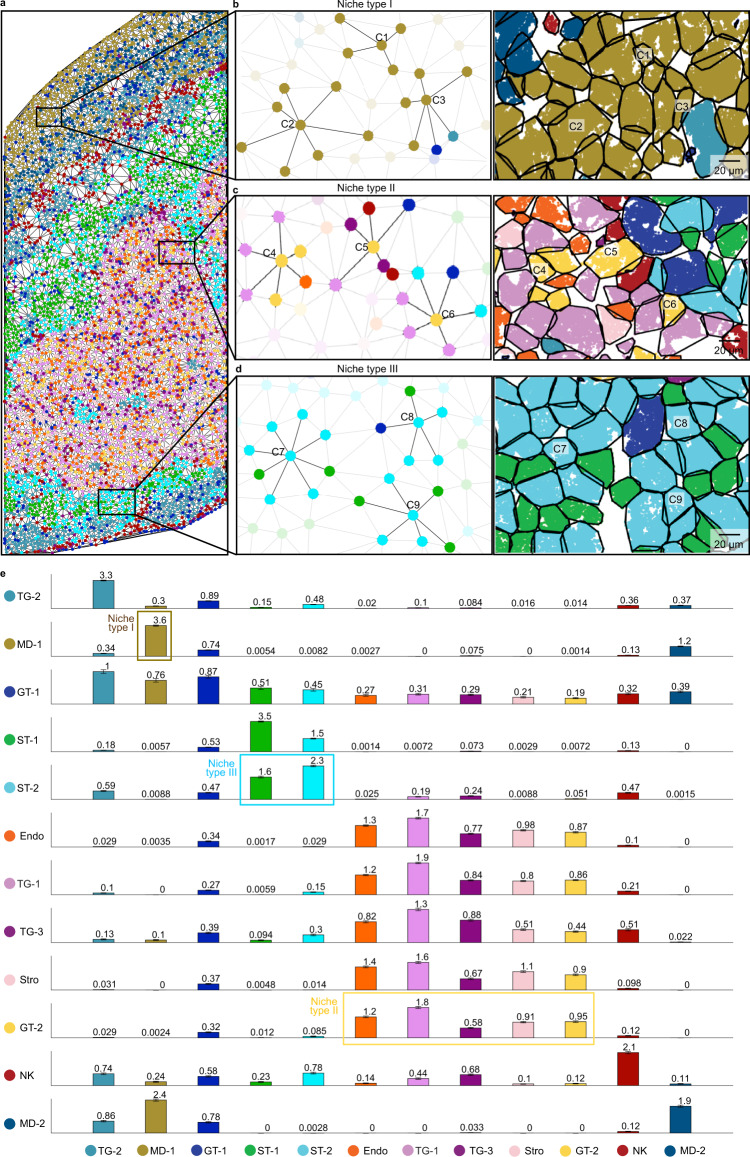
ClusterMap reveals cell niche and cell–cell adjacency graph in the placenta. **a** Mesh graph generated by Delaunay triangulation^26^ of cells shown in the STARmap mouse placenta 903-gene reveals cell niche. Each cell is represented by a spot in the color of its corresponding cell type. Physically neighboring cells are connected via edges. **b**–**d** A zoomed-in view of the top, middle, and bottom square in **a**. The intercellular connection is centered on three MD-1 type (C1, C2, C3), GT-2 type (C4, C5, C6), and ST-2 (C7, C8, C9) type cells, respectively, with their first tier of neighboring cells highlighted. Left: schematic; right: cell segmentation map. **e** Bar plots of the average number of cells per cell type among the first-tier neighbors, revealing clear patterns of cell-type specific cell–cell communication. Cells in Niche Type I, II, and III show selective association with cell types highlighted in the corresponding bounding box. The cell types on the axes are denoted by initializes. Data are presented as mean values ± SEM.

We further sought to use ClusterMap results to characterize the near-range cell adjacency networks by generating a mesh graph via Delaunay triangulation of cells and modeling the cellular relationships based on the i-niche concept^26^. In this way, we identified the nearest neighbors of each cell which were directly contacting each other (Fig. 4a–d) and quantified the average number of cells per cell-type among the first-tier neighbors (Fig. 4e), which could reveal crucial information about the affinity and communication among different cell types. Through this methodology, we discovered the cell-type-specific cellular adjacency graph: MD-1, trophoblast giant-2 (TG-2), and NK cells mainly self-aggregate; glandular trophoblast-2 (GT-2), TG-1, TG-3, endothelial and stromal cells widely connect with these five types of cells; and Spongiotrophoblast -1 and Spongiotrophoblast -2 cells have a high affinity to each other. To further explore if cell niche influences gene expression and further defines cell subtypes, as an example, we sub-clustered MD-1 cells based on either gene expression (Louvain clustering) or the cell niche compositions (*K*-means clustering). Both subclustering results identified two subtypes. Confirming the similarity between two subclustering results by adjusted Rand index (ARI) (ARI = 0.62, Supplementary Fig. 7 and Methods section) suggests that cell adjacency graph analysis can help identify subtypes shaped by cell niche. We envision that identifying the cell-cell adjacency graph facilitates future in-depth studies of tissue architecture.

### ClusterMap is applicable across various in situ transcriptomic methods

**Fig. 5:**
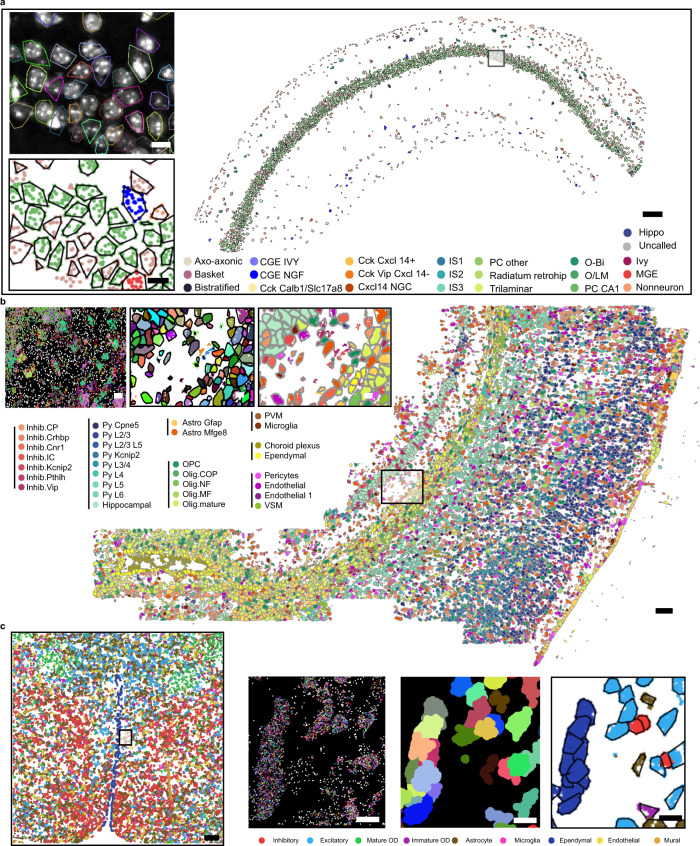
ClusterMap across different spatial transcriptomics methods. **a** Cell type map of the pciSeq (ISS data) section 4–3 left CA1 dataset^4^. Scale bar: 200 µm. Insets from top to bottom: convex hull of ClusterMap-identified cells overlapped with the DAPI image and zoom-in cell type map in the black box highlighted region. Scale bar: 10 µm. **b** Cell type map of whole osmFISH mouse SSp datasets^5^. Scale bar: 100 µm. Insets from left to right: raw spatial transcriptomics data, and corresponding cell segmentation map and cell type map of the black box highlighted region. Scale bar: 10 µm. **c** The 2D cell type map of whole MERFISH mouse POA datasets^3^. Scale bar: 200 µm. Insets from left to right insets: 2D raw spatial transcriptomics data, and corresponding cell segmentation map and cell type map of the highlighted region. Scale bar: 10 µm.

Beyond STARmap^6^, we further applied ClusterMap to analyze mouse brain tissue from three other in situ transcriptomics methods. Analyses of the imaged transcripts in 2D mouse hippocampal area CA1 by pciSeq (ISS data)^4^, 2D somatosensory cortex by osmFISH^5^, and 3D hypothalamic preoptic tissues by MERFISH^3^ are shown respectively in Fig. 5. We used RNA spot matrices from the published data^3–5^ and applied ClusterMap analysis described in Fig. 1b. Despite the differences in experimental designs and the number of transcript copies across protocols, ClusterMap identifies cells successfully. As an example, the ClusterMap-identified cell boundaries over the DAPI image show accurate cell segmentations in ISS CA1 datasets^4^ (Fig. 5a). In all three datasets, the identified cell types and their spatial patterns from ClusterMap were consistent with published results from conventional segmentation methods or scRNA-seq (Fig. 5 and Supplementary Fig. 8). Specifically, for ISS data of the mouse hippocampus, we further conducted tissue region segmentation and provided detailed statistics of cell type percentage of each region (Supplementary Fig. 9). We observed that the fine cell classes of the CA1 region displayed distinct laminar locations, and pyramidal cells account for 89% cells in the whole CA1 soma region, which are consistent with results in pciSeq. Notably, ClusterMap can provide more detailed cell morphology, increased number of cells, and increased number of total reads (Supplementary Fig. 8). In conclusion, we analyzed mouse brain data from four representative in situ transcriptomic methods^3–6^ and validated the general applicability of ClusterMap for different experimental methods with negligible modification applied.

### 3D ClusterMap analyses in thick tissue blocks

**Fig. 6:**
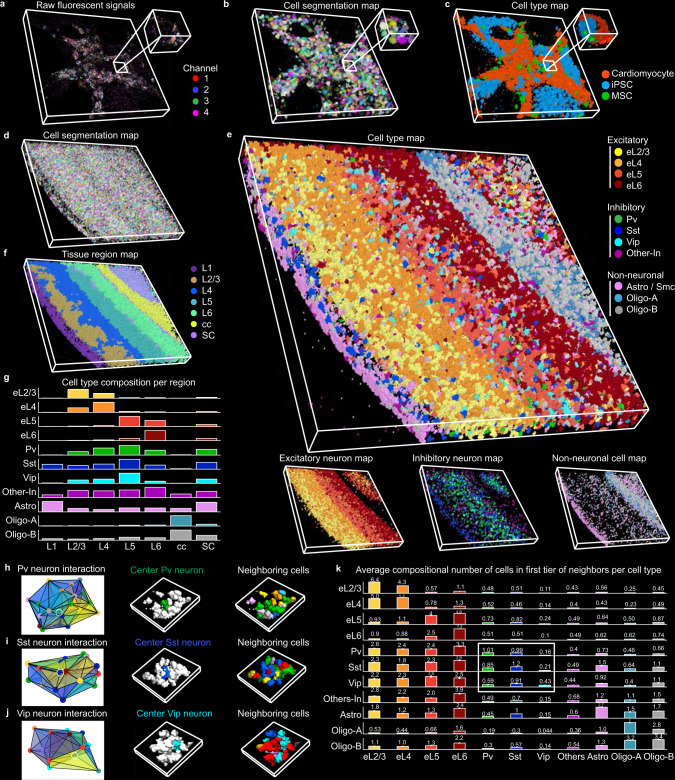
ClusterMap enables 3D in situ transcriptomics analysis. **a** Raw fluorescent signals of 3D STARmap cardiac organoid 8-gene dataset. Width: 465 µm, height: 465 µm, depth: 97 µm. **b**, **c** ClusterMap generates 3D cell segmentation map (**b**) and cell-type map (**c**) of (**a**), which includes 1519 cells. Insets in (**a**–**c**) show zoomed-in views of the highlighted regions. **d** ClusterMap generates a volumetric cell segmentation map of 3D STARmap mouse V1 28-gene dataset^6^, showing 24,590 cells. Width: 1545 µm, height: 1545 µm, depth: 100 µm. **e** The 3D cell type maps of **d** show the spatial cell type distribution. **f** The 3D tissue region map of (**e**). SC, subcortical. **g** Bar plots of composition of 11 cell types across 7 tissue regions (layers). **h**–**j** Example of cellular communication at a Pv, Sst, or Vip neuron, respectively. Left: schematics of 3D Delaunay triangulation of the Pv, Sst, or Vip neuron (highlighted in a white circle) and its first tier of neighboring cells. Middle: 3D spatial cell distribution of the first panel with the first tier of neighboring cells colored in white. Right: 3D spatial cell distribution of the first panel. Width 184 µm, height 194 µm, depth 100 µm. **k**, Bar plots of average composition of cell types around each cell type. Patterns of self-association in the minority inhibitory neurons are highlighted in the bounding box. Cell types in **g**–**k** are color-coded as in **e**. Data are presented as mean values ± SEM.

3D in situ transcriptomics data analysis is considered even more challenging because it is generally infeasible by manual labeling. However, 3D volumetric imaging and analysis are required to understand the structural and functional organization of complex organs. In this regard, exploring ClusterMap’s ability to analyze 3D in situ transcriptomics is particularly desired. We applied ClusterMap to two 3D thick-tissue samples: STARmap cardiac organoid 8-gene dataset^27^ and STARmap mouse V1 28-gene dataset^6^ (Supplementary Table 1). We analyzed the 3D data following the sample protocol described in Fig. 1b. In the 3D cardiac organoid sample, hierarchical clustering^28^ separated cells into three categories with distinct molecular signatures (Fig. 6a–c): *CD44* for mesenchymal stem cells (MSCs), *Nanog* for induced pluripotent stem cells (iPSCs) and four genes (*TNNI1*, *MYH7*, *MYL7*, *ATP2A2*) for cardiomyocytes (Supplementary Fig. 10a–c). The 100-μm-thick sample of mouse V1 includes all six cortical layers and the corpus callosum, in which up to 24,000 cells were identified and 3D clustered into eleven cell types (Fig. 6d, e and Supplementary Fig. 10d–g). Our results showed similar spatial distribution with previously published results, which used the conventional fluorescence image segmentation: excitatory neurons exhibited a gradient distribution, with the spatial density of each subtype gradually decaying to adjacent layers across the entire 3D space; inhibitory neurons showed a more dispersed distribution; and non-neuronal cells were largely located in the white matter and layer 1 (Fig. 6e). We can determine seven 3D tissue regions based on their corresponding cell-type compositions (Fig.6f, g). We further characterized 3D cell-cell niche in the mouse V1 and computed the average compositional neighboring cell types (Fig. 6h–k). In the minority inhibitory neurons, we observed a similar self-associative pattern as in previously published findings^6^: the nearest neighbor of any inhibitory neuron tends to be its own subtype. Three adjacency graph examples of inhibitory neuronal types (Pv, Sst, Vip) are presented in Fig. 6h–j, respectively.

## Discussion

Spatial RNA localization intrinsically contains information related to biological structures and cell functions. ClusterMap exemplifies a computational framework that combines spatial and high-dimensional transcriptomic information from in situ single-cell transcriptomics to identify subcellular, cellular, and tissue structures in both 2D and 3D space. Clustermap jointly clusters the physical density and gene identity of RNAs, which provides higher accuracy than clustering only using RNA density or gene identity (Supplementary Fig. 11). Compared with previous methods^20^ (Supplementary Figs. 1, 2, and 12), ClusterMap showed consistently high performance in both simulated and biological datasets. In addition, ClusterMap is widely applicable to various experimental methods including, but not limited to, STARmap^6^, MERFISH^3^, ISS^4^, and osmFISH^5^. As a result, ClusterMap accurately created RNA-annotated subcellular and cellular atlases from in situ transcriptomic data across diverse tissue samples with different RNA localization, cell density, morphologies and connections. This will markedly expand our knowledge of cellular organization across all scales from subcellular organelles through cell-type maps to organs and enable further characterization of the local microenvironment for individual cells. Our initial successful demonstration suggests that in situ transcriptomic profiles contain unexplored biological and structural information that can be further extracted by new computational strategies.

Beyond spatial transcriptomic data, ClusterMap can be generalized and applied to other 2D and 3D mapped high-dimensional discrete signals (e.g., proteins or live-cell imaging data)^29^. In the future, we envision that ClusterMap can also be extended by combining other types of biological features (e.g., subcellular organelles, cell shapes, etc.) to uncover the basic principles of how gene expression shapes cellular architecture and tissue morphology^30^.

## Methods

### Thin-section STARmap data pre-processing

All image processing steps^31–45^ were implemented using MATLAB R2019b and related open-source packages in Python 3.6 according to Wang et al.^6^.

#### Image preprocessing

For better unity of the illuminance and contrast level of the raw fluorescence image, a multi-dimensional histogram matching was performed on each image, which used the image of the first color channel in the first sequencing round as a reference.

#### Image registration

Global image registration for aligning spatial position of all amplicons in each round of STARmap imaging was accomplished using a three-dimensional Fast Fourier transform (FFT) to compute the cross-correlation between two image volumes at all translational offsets. The position of the maximal correlation coefficient was identified and used to transform image volumes to compensate for the offset.

#### Spot finding

After registration, individual spots were identified separately in each color channel on the first round of sequencing. For this experiment, spots of ~6 voxels in diameter were identified by finding local maxima in 3D. After identifying each spot, the dominant color for that spot across all four channels was determined on each round in a 5 × 5 × 3 voxel volume surrounding the spot location.

#### Spots and barcode filtering

Spots were first filtered based on fluorescence quality score. Fluorescence quality score is the ratio of targeted single-color channel to all color channels, which quantified the extent to which each spot on each sequencing round came from one color rather than a mixture of colors. Each spot is assigned with a barcode representing a specific kind of gene. The barcode codebook that contains all gene barcodes was converted into color space, based on the expected color sequence following 2-base encoding of the barcode DNA sequence^6^. Spot color sequences that passed the quality threshold and matched sequences in the codebook were kept and identified with the specific gene that that barcode represented; all other spots were rejected. The high-quality spots and associated gene identities in the codebook were then saved out for downstream analysis.

#### 2D manual cell segmentation

Two different methods were used to identify cell boundaries. First, the manually labeled segmentation masks from the original reference (Wang et al.^6^) were obtained as baseline. Second, nuclei were automatically identified by the StarDist 2D machine learning model (Schmidt et al.^15^) from a maximum intensity projection of the DAPI channel following the final round of sequencing. Then cell locations were extracted from the segmented DAPI image. Cell bodies were represented by the overlay of DAPI staining and merged amplicon images. Finally, a marker-based watershed transform was then applied to segment the thresholded cell bodies based on the combined thresholded cell body map and identified locations of nuclei. For each segmented cell region, a convex hull was constructed. Points overlapping each convex hull in 2D were then assigned to that cell, to compute a per-cell gene expression matrix.

### Thick-tissue STARmap data pre-processing

#### 3D image registration

The displacement field of each imaging round was first acquired by registering the DAPI channel of each round to first-round globally by 3D FFT. Each sequencing image was applied with the corresponding transform of its round.

#### Spot finding

After registration, individual spots were identified separately in each color channel on each round of sequencing. The extended local maxima in 3D were treated as an amplicon location. After identifying each spot, the dominant color for that spot across all four channels was determined on each round in a 3 × 3 × 3 voxel volume surrounding the spot location.

### Computation of neighborhood gene composition

To compute the NGC composition of each spot, we considered a spatially circular (2D) or spherical (3D) window over every spot (*S*) and counted the number of each gene-type among spots in the window. The radius of the window *R* can be chosen either manually or by statistics close to the averaged size of organelles and cells for subcellular and single cell analyses, respectively.

In a dataset with *T* kinds of sequenced genes, the definition of an NGC vector for a measured spot *i* is the number of each gene-type windowed by radius *R* to the measured spot *i*.

1 $${NGC}\left(i\right)= < {{Num}}_{{Gene}\ 1},{{Num}}_{{Gene}\ 2},\ldots ,{{Num}}_{{Gene}\ t},\ldots ,{{Num}}_{{Gene}\ T} > $$

2 $${{Num}}_{{Gene}\ t}=\#\{{S}_{t}^{1},{S}_{t}^{2},\ldots ,{S}_{t}^{j},\ldots ,{S}_{t}^{{{Num}}_{{Gene}\ t}}\},t\in {N}^{T}$$

3 $${Distance}\{{S}_{t}^{j},i\} \, < \, R,t\in {N}^{T},j\in {N}^{{{Num}}_{{Gene}\ t}}$$

### Density peak clustering (DPC)

Based on the original DPC algorithm^18^, we first computed the two quantities: local density *ρ* and distance *δ* of every spot. We estimated the density by a Gaussian kernel with variance *d*_*c*_. The variance *d*_*c*_ is supposed to be close to the averaged radius *R* of cells for cellular segmentation. We can use *R* as *d*_*c*_. The definition of local density *ρ* and distance *δ* for spot *i* is:

4 $${\rho }_{i}=\mathop{\sum} _{j}I({d}_{{ij}}-{d}_{{\max }})\ast {e}^{-{({d}_{{ij}}/R)}^{2}}$$

5 $${\delta }_{i}={min }({d}_{{ij}}),\ j{{{{{\rm{:}}}}}}\ {\rho }_{j} \, > \, {\rho }_{i}$$

Note that *I*(*x*) = 1 *if x* < 0, *else I*(*x*) = 0, and *d*_*ij*_ is the distance between spot *i* and *j*. The optional parameter *d*_*max*_ is a restriction on the maximum radius of the cell. For the point with the highest density, based on principles of DPC^18^, we took its distance value to the highest *δ* value. Note that for large data sets, the analysis is insensitive to the choice of *d*_*c*_ and results are robust and consistent^18^.

After computing these two quantities for spots, we generated a multiplication decision graph by computing *γ*, the product of *ρ* and *δ* and plotting every spot’s *γ* value in decreasing order. Since the cell centers have both high local density and much higher distance at the same time, we chose the points with distinguishably higher *γ* values as cluster centers. We chose the ‘elbow point’ as the cutoff point in the multiplication decision graph where the *γ* value becomes no longer high and the change tends to be flat. The number of clusters *N* is equal to the number of points prior to the elbow point.

Next, we assigned each remaining point to one of the *N* clusters respectively in a descending order of *ρ* value in a single step manner. Each remaining spot was assigned to the same cluster as its nearest cluster-assigned neighbor. Each cluster was regarded as one cell. Finally, we filtered cells by limiting the minimum number of spots and genes expressed in one cell.

### Integration of the physical and NGC coordinate

The physical coordinates denote the spatial location of spots and the NGC coordinates denote the gene location of spots in a high-dimensional NGC space. For spot *i*, its physical and NGC coordinate are:

6 $$P\left(i\right)= < {x}_{i},{y}_{i},({z}_{i}) > $$

7 $${NGC}\left(i\right)= < {{Num}}_{{Gene}\ 1},{{Num}}_{{Gene}\ 2},\ldots ,{{Num}}_{{Gene}\ t},\ldots ,{{Num}}_{{Gene}\ T} > $$

#### Distance-level integration

We computationally integrated the NGC and physical coordinates into the joint P-NGC coordinate over each spot. Here, to apply the density peak clustering algorithm, we used inversed Spearman correlation coefficient to measure the distance between two NGCs, and combined the physical and NGC distances information between *i* and its neighboring spots. We used the joint distance as the metric to measure relationships between spots. Mathematically, the parameter *d*_*ij*_ used in the calculation of *ρ* and *δ* in DPC is:

8 $${d}_{{ij}}=\frac{{{{Distance}}}\{{P\left(i\right),P(j)}\}}{{{{SpearmanCorr}}}{\{{{{{{{\mathrm{NGC}}}}}}}\left(i\right),{{{{{{\mathrm{NGC}}}}}}}(j)\}}}$$

Then we used the combined distances to perform the DPC algorithm for cell segmentation. Note that sometimes the inconsistency of spot relationships between physical distance and Spearman correlation may break the physical connectivity of spots within one cell. In this case, a 0.5 lower boundary cutoff may be applied to correlation values. Also, we modified the DPC algorithm implementation by using joint distances to find cell centers and then physical distances to assign other spots to cell centers to preserve the physical connectivity of cells. This integration method is universal to any datasets.

### Pre- and post-processing for quality control

First, a background identification step to filter input spots was used as pre-processing. Specifically, regions with low-density spots (mRNA or DAPI sampled spots) are considered as noisy background that will be removed for the downstream analysis. Second, the noise rejection based on cluster halo (i.e. noise) identification in the original density peak clustering algorithm^18^ was used as post-processing. Specifically, instead of introducing a noise-signal cutoff, we first found a border region for each cell, then identified the point of highest density of spots (mRNA or DAPI sampled spots) within its border region as *ρ*_*b*_, and finally considered points within the cell that show higher density than *ρ*_*b*_ as the robust assignment for spots in border region and others as noise. These quality control steps have been included in the analysis of three representative in situ transcriptomic datasets^3–5^ (Fig. 5).

### Subcellular segmentation

To perform subcellular segmentation and construct nuclear boundaries we first computed the quantity NGC over each spot in an individual cell. The difference between NGC for subcellular segmentation and that for cellular segmentation is the radius of the window *R*. *R* should be either chosen manually or by statistics to be close to the averaged size of organelles. In addition, when the number of sequenced genes is limited, we can compute the NGC using a mesh graph by Delaunay triangulation of spots that models the relationship between RNA spots in the cell. A ring of spots that are neighbors of the central spot in the mesh graph is considered to locate most closely around the central spot. For a dataset with *TR* kinds of gene the definition of an NGC vector to the measured spot *i* is the composition of gene-types in its closest neighbors:

9 $${NGC}\left(i\right)= < {{Num}}_{{Gene}\ 1},{{Num}}_{{Gene}\ 2},\ldots ,{{Num}}_{{Gene}\ t},\ldots ,{{Num}}_{{Gene}\ {TR}} > $$

10 $${{Num}}_{{Gene}\ t}=\#\{{S}_{t}^{1},{S}_{t}^{2},\ldots ,{S}_{t}^{j},\ldots ,{S}_{t}^{{{Num}}_{{Gene}\ t}}\},t\in {N}^{{TR}}$$

$${S}_{t}^{j}\,{connects}\,{directly}\,{with}\,{spot}\,i,\forall j\in {N}^{{{Num}}_{{Gene}\ t}}\left.\right\},$$

Then, similar to distance-level integration, we generated a joint P-NGC coordinate from the normalized NGC and physical coordinates over each spot:

11 $$P-{NGC}\left(i\right)=[{NGC}\left(i\right),\lambda \ast P(i)]$$

Here the optional parameter *λ* can control the influence of physical coordinates, depending on conditions. We then used *K*-means clustering^19^ to cluster spots into two regions with one for nucleus and one for cytoplasm. Under a chosen *λ*, *K*-means clustering was performed 100 times with different seed each time to find the consensus clustering results. Finally, we constructed a convex hull based on the nucleus spots, denoting the nuclear boundary.

### Cell type classification

For datasets STARmap mouse V1 1020-gene and STARmap mouse V1 28-gene, a two-level clustering strategy was applied to identify both major and sub-level cell types. Processing steps in this section were implemented using Scanpy v1.6.0 and other customized scripts in Python 3.6 and applied according to Wang et al., 2018^6^. After filtration, normalization, and scaling, principal-components analysis (PCA) was applied to reduce the dimensionality of the cellular expression matrix. Based on the explained variance ratio, the top PCs were used to compute the neighborhood graph of observations. Then the Louvain algorithm^22^ was used to identify well-connected cells as clusters in a low dimensional representation of the transcriptomics profile. Clusters enriched for the excitatory neuron marker *Slc17a7* (vesicular glutamate transporter), inhibitory neuron marker *Gad1*, were manually merged to form two neuronal cell clusters, and then other cells represented non-neuronal cell populations. The cells were displayed using the uniform manifold approximation and projection (UMAP) and color-coded according to their cell types. The cells for each top-level cluster were then sub-clustered using PCA decomposition followed by Louvain clustering^22^ to determine sub-level cell types. For dataset pciSeq mouse CA1, the probabilistic model in pciSeq^4^ is used to assign ClusterMap-identified cells to scRNA seq data and find cell-types. For dataset MERFISH mouse POA and osmFISH mouse SSp, hierarchical clustering is applied to find cell types that match previous reported cell types. For other datasets, Louvain clustering algorithm is applied to find cell types.

### Construct tissue regions

#### Neighborhood Cell-type Composition (NCC)

To construct tissue regions, we computed a global quantity: Neighborhood Cell-type Composition (NCC) over each cell (*C*). We considered a spatially circular (2D) or spherical (3D) window over every cell and estimated the composition of cell-types in the window. The radius of the window RC was chosen manually or by statistics of distances between cells to be as reasonable as possible.

For a dataset with *TC* kinds of gene, the definition of an NCC vector of the measured cell *i* was the composition of cell-types in the defined window that had radius RC to the measured cell *i*.

12 $${{{NCC}}}\left(i\right)= < {{{{Num}}}}_{{{{Cell}}}{{{type}}}\ 1},{{{{Num}}}}_{{{{Cell}}}{{{type}}}\ 2},\ldots ,{{{{Num}}}}_{{{{Cell}}}{{{type}}}\ t},\ldots ,{{{{Num}}}}_{{{{Ce}}}{{{ll}}}{{{type}}}\ {TC}} > $$

13 $${{{{Num}}}}_{{{{Cell}}}{{{type}}}\ t}=\#\{{C}_{t}^{1},{C}_{t}^{2},\ldots ,{C}_{t}^{j},\ldots ,{C}_{t}^{{{{{{{{\mathrm{Num}}}}}}}}_{{{{{{Cell}}}}}{{{{{type}}}}}\ t}}\},t\in {N}^{{TC}}$$

14 $${{{Distance}}} \{{C}_{t}^{j},i\} \, < \, {RC},t\in {N}^{{TC}},j\in {N}^{{{{{{{{\mathrm{Num}}}}}}}}_{{{{{{Cell}}}}}{{{{{type}}}}}\ t}}$$

#### *K*-means clustering

Tissue region signatures were identified using information from both NCC and physical locations of cells. Then we generated a joint P-NCC coordinate from normalized NCC and physical coordinates over each cell:

15 $${P}-{{{NCC}}}\left(i\right)=[{{{NCC}}}\left(i\right),\lambda \ast P(i)]$$

Here the optional parameter *λ* can control the influence of physical coordinates based on conditions. We then used *K*-means clustering on these high dimensional P-NCC coordinates to cluster cells into a pre-defined number of regions. Under a chosen *λ*, *K*-means clustering was performed 100 times with different seed each time, and the most frequent clustering results with interpretable biological meanings was regarded as final clustering. Finally, we projected spatially back onto the cell-type map.

### Compare with expert-annotated labels

We evaluated the accuracy of cell identification by ClusterMap with corresponding eight expert annotated STARmap^6^ datasets (Supplementary Fig. 3c). Cells defined by ClusterMap consist of spots with physical locations while labels in the expert annotated STARmap datasets are connected components. We defined the accuracy as the percentage of ClusterMap-identified cells that correctly matched the manual labeled cells. Specifically, for each labeled connected component, we checked if there was only one predicted cell by ClusterMap within the region. More than one cell was counted as over-segmentation and no cell as under-segmentation.

We also compared the correlation of the single-cell gene expression profiles between ClusterMap and expert-annotated labels in STARmap^6^ mouse V1 1020-gene (Supplementary Fig. 5a, b). For the shared 13 cell types identified in cells from both ClusterMap and manual annotation, we computed the average gene expression values across 1020 genes. Then we calculated the Pearson correlation and *p*-value between two cell-type-by-gene-expression matrices and plotted as heatmaps in Supplementary Fig. 5. We observed high correlation values and low p-values in matched cell types in between ClusterMap and expert-annotated labels, which further validated the performance of ClusterMap.

### Performance analysis of cell segmentation in ClusterMap

We further evaluated the performance of ClusterMap using the following three conditions: (1) only physical distances, (2) only neighborhood gene composition (NGC) distances, and (3) joint physical and NGC distances from published STARmap V1 1020-gene datasets^6^ with ground truth labels in Supplementary Fig. 11a–e. The results show that solely using physical distance or NGC distance for cell segmentation, ClusterMap is less effective when there is a lack of RNA signals in nuclei or when cells are crowded as shown in Supplementary Fig. 11a. ClusterMap with an integrative physical and NGC information can overcome these issues and provide a better cell segmentation, with lower under-/over- segmentation scores and higher accuracy (Supplementary Fig. 11a–c). To further examine and highlight the difference, we built the toy model by assigning random gene identities (Supplementary Fig. 11d) or identical gene identities (Supplementary Fig. 11e) to RNA spots and then tested the performance of ClusterMap by using the aforementioned three conditions. As shown in Supplementary Fig. 11d, e, the results further support our conclusion that gene identity is important to generate a more accurate cell segmentation result. In conclusion, ClusterMap incorporates physical and neighborhood gene expression information to improve cell segmentation performance.

We provided performance analysis of ClusterMap cell segmentation in mouse placenta tissue where the cells were of vastly different sizes and shape, and cell radius *d*_c_ ranged from 28 to 128 pixels (2.65–12.12 µm) (Supplementary Fig. 11f). With the radius used in ClusterMap increasing from 8 to 178 pixels, the number of cells decreased from 270 to 220. The accuracy increased first as the radius increased from 8 to 28 pixels, then remained relatively stable, and finally dropped when the radius exceeded 148 pixels (Supplementary Fig. 11g, h). The radius of 83 pixel with the highest accuracy was checked to be a frequent radius for most cells.

Finally, we showed that in the cases when RNAs populate nucleus and cytoplasm, incorporation of DAPI signal will improve the performance of ClusterMap. We tested on STARmap mouse V1 1020-gene datasets where thousands of genes have been in situ sequenced and RNA is enriched in the nucleus (Supplementary Fig. 11i, l). Two examples of the hippocampus regions comparing the performance of ClusterMap with and without DAPI signal input are shown in Supplementary Fig. 11i–n. The results show that the integration of DAPI signals with RNA signals substantially decreased the percentage of over-/under- segmented cells and improved accuracy from 0.75 to 0.81 (Supplementary Fig. 11o, p).

### Label transfer

Cell type labels from scRNA-Seq dataset were projected onto spatially resolved cells from STARmap dataset by using the Seurat v3 integration method according to Stuart et al.^24^. First, both datasets were preprocessed (normalization and scaling) and a subset of features (e.g., genes) exhibiting high variability was extracted. For STARmap dataset, all genes profiled were used whereas in scRNA-Seq dataset, the top 2,000 most variable genes identified by “*FindVariableFeatures*” function were used in downstream integration. Then “*FindTransferAnchors*” (reduction = “cca”) and Transfer Data functions were used to map the labels onto spatially resolved cells from the STARmap dataset. After label transferring, 6672 out of 7224 cells were observed with high-confidence cell type predictions (prediction score >0.5), and 8 cell types labels were resolved.

### Sub-clustering by cell niche analysis

Sub-clustering cell types in STARmap mouse placenta 903-gene dataset: First, for 7224 ClusterMap-identified cells, we constructed two matrices: (1) cell by gene matrix, which is 7224 × 903 dimensions; (2) cell by cell niche composition matrix, which is 7224 × 12 dimensions. Next, for *N* cells of a certain cell type *T*, we got a *N* × 903 subset matrix and a *N* × 12 subset matrix, which provided gene expression and cell niche composition information about the *N* cells. Then, Louvain clustering was used to cluster the *N* × 903 gene expression matrix into *S* sub-types, and *K*-means clustering was used to cluster the *N* × 12 cell niche composition matrix into *S* sub-types. Finally, *N* cells were mapped to UMAP based on their gene expression and are colored based on two data clustering. Adjusted Rand index of two data clustering was computed.

### Statistics and reproducibility

In Fig. 2e, the number of cells per cell type in each region are as follows: from L1 to HPC, eL2/3-A: 3, 164, 22, 12, 6, 1, 0; eL2/3-B: 0, 33, 4, 3, 2, 0, 0, 0;eL4: 0, 7, 135, 7, 0, 0, 0; eL5: 0, 1, 9, 62, 39, 2, 5; eL6: 0, 1, 0, 19, 133, 0, 2; Hpc: 0, 0, 0, 1, 0, 0, 9; Pv: 0, 7, 7, 16, 5, 0, 2; Vip: 4, 15, 2, 2, 1, 1, 2; Sst: 0, 6, 6, 13, 3, 0, 12; Others-In: 0, 0, 3, 6, 1, 2, 6; Astro: 7, 24, 12, 24, 14, 19, 21; Endo: 9, 39, 25, 30, 16, 3, 12; Micro: 6, 20, 6, 12, 3, 8, 7; Other: 4, 41, 22, 50, 20, 6, 7; Oligo: 1, 5, 7, 23, 13, 100, 15; and Smc. 10, 0, 0, 1, 0, 0, 1. In Fig. 3h, the number of cells in each region is as follows: I: 1457; II: 1796; III: 2816; IV: 777; and V: 378. In Fig. 6g, the number of cells in each cell type is as follows: Cardiomyocytes, 929; induced pluripotent stem (iPS) cells, 489; and mesenchymal stem cells (MSC), 101. In Fig. 6g, the number of cells per cell type in each region are as follows: from L1 to SC, eL2/3: 31, 1767, 965, 119, 113, 9, 173; eL4: 16, 722, 1596, 168, 89, 4, 136; eL5: 5, 39, 92, 1000, 596, 6, 202; eL6: 11, 74, 191, 541, 2500, 18, 550; Pv: 4, 6, 136, 183, 94, 7, 111; Sst: 97, 72, 101, 196, 81, 11, 136; Vip: 3, 22, 28, 66, 14, 1, 28; Other-In: 30, 78, 74, 83, 112, 39, 68; Astro: 275, 92, 65, 106, 104, 92, 256; and Oligo-A: 28, 33, 33, 80, 95, 1014,183; Oligo-B: 81, 63, 86, 158, 131, 536, 257. In Supplementary Fig. 3c, the number of manual annotated cells in each sample are as follows: BZ5: 1227; BZ9: 1318; BZ14:1203; BZ19: 1370; BD2: 951; BD6: 788; BY1: 1653; BY3:1008.

### Animal experiment

C57BL/6 (female, 8–12 weeks) mice were purchased from the Jackson Laboratory (JAX). Animals were housed 2–5 per cage and kept on a reversed 12 h light-dark cycle with ad libitum food and water. For the mouse placenta dataset, we used snap-frozen tissue sections from C57BL/6 J x CAST/EiJ matings and performed STARmap to measure expression of 903 genes on the E14.5 mouse placenta tissue slices. Sex: female. Age: E14.5. Strain: C57BL/6 J x CAST/EiJ matings. Housing conditions: Mice were housed under standard barrier conditions at the Whitehead Institute for Biomedical Research. All procedures involving animals at the Broad Institute were conducted in accordance with the US National Institute of Health Guide for the Care and Use of Laboratory Animals under protocol number 0255-08-19. Experimental procedures were approved by the Institutional Animal Care and Use Committee of the Broad Institute of MIT and Harvard under protocol number 0255-08-19.

### Reporting summary

Further information on research design is available in the Nature Research Reporting Summary linked to this article.

## Supplementary information

Supplementary Information

Reporting Summary

## Data Availability

The MERFISH mouse POA set^3^, osmFISH mouse SSp set^5^, and pciSeq mouse isocortex set^4^ are available in Supplementary Information. MERFISH mouse POA set is at http://zhuang.harvard.edu/merfish.html, osmFISH mouse SSp set is at http://linnarssonlab.org/osmFISH, and pciSeq mouse hippocampus set is at https://figshare.com/s/88a0fc8157aca0c6f0e8. The STARmap mouse V1 1020-gene, STARmap mouse V1 28-gene set, STARmap cardiac organoid set and STARmap mouse placenta are available at Code Ocean^46^.

## Code availability

ClusterMap is implemented based on MATLAB R2019b and Python 3.6. The following packages and software were used in data analysis: UCSF ChimeraX 1.0, ImageJ 1.51, MATLAB R2019b, R 4.0.4, Rstudio 1.4.1106,, Jupyter Notebook 6.0.3, Anaconda 2-2-.02, h5py 3.1.0, hdbscan 0.8.36, hdf5 1.10.4, matplotlib 3.1.3, seaborn 0.11.0, scanpy 1.6.0, numpy 1.19.4, scipy 1.6.3, pandas 1.2.3, scikit-learn 0.22, umap-learn0.4.3, pip 21.0.1, numba 0.51.2, tifffile 2020.10.1, scikit-image 0.18.1, itertools 8.0.0. Codes are available at Code Ocean https://codeocean.com/capsule/6072400/^46^. The ClusterMap tool will be maintained and updated at https://github.com/wanglab-broad/ClusterMap and https://github.com/LiuLab-Bioelectronics-Harvard/ClusterMap.
