# Supplementary material for: ClusterMap for multi-scale clustering analysis of spatial gene expression
Source: Nat Commun. 2021 Oct 8;12:5909. doi: 10.1038/s41467-021-26044-x (PMC8501103; doi:10.1038/s41467-021-26044-x)
Supplement: Supplementary file 1 — Supplementary Information [file 41467_2021_26044_MOESM1_ESM.pdf]

Supplementary Information

ClusterMap for multi-scale clustering analysis of spatial gene expression

Yichun He[1,2,8], Xin Tang[1,2,8], Jiahao Huang[2], Jingyi Ren[2,3], Haowen Zhou[2], Kevin Chen[4], Albert Liu[2,3], Hailing Shi[2,3], Zuwan Lin[4,2], Qiang Li[1], Abhishek Aditham[2,5], Johain Ounadjela[2,6], Emanuelle I Grody[2,6], Jian Shu[2,6,7], Jia Liu[1*] & Xiao Wang[2,3*]

[1]John A. Paulson School of Engineering and Applied Sciences, Harvard University, Cambridge, MA, USA.

[2]Broad Institute of MIT and Harvard, Cambridge, MA, USA.

[3]Department of Chemistry, Massachusetts Institute of Technology, Cambridge, MA, USA.

[4]Department of Chemistry and Chemical Biology, Harvard University, Cambridge, MA, USA.

[5]Department of Biological Engineering, Massachusetts Institute of Technology, Cambridge, MA, USA.

[6]Whitehead Institute for Biomedical Research, Cambridge, MA, USA.

[7]Cutaneous Biology Research Center, Massachusetts General Hospital, Harvard Medical School, Boston, MA, USA.

[8]These authors contributed equally: Yichun He, Xin Tang.

[*]e-mail: wangxiao@broadinstitute.org; jia_liu@seas.harvard.edu.

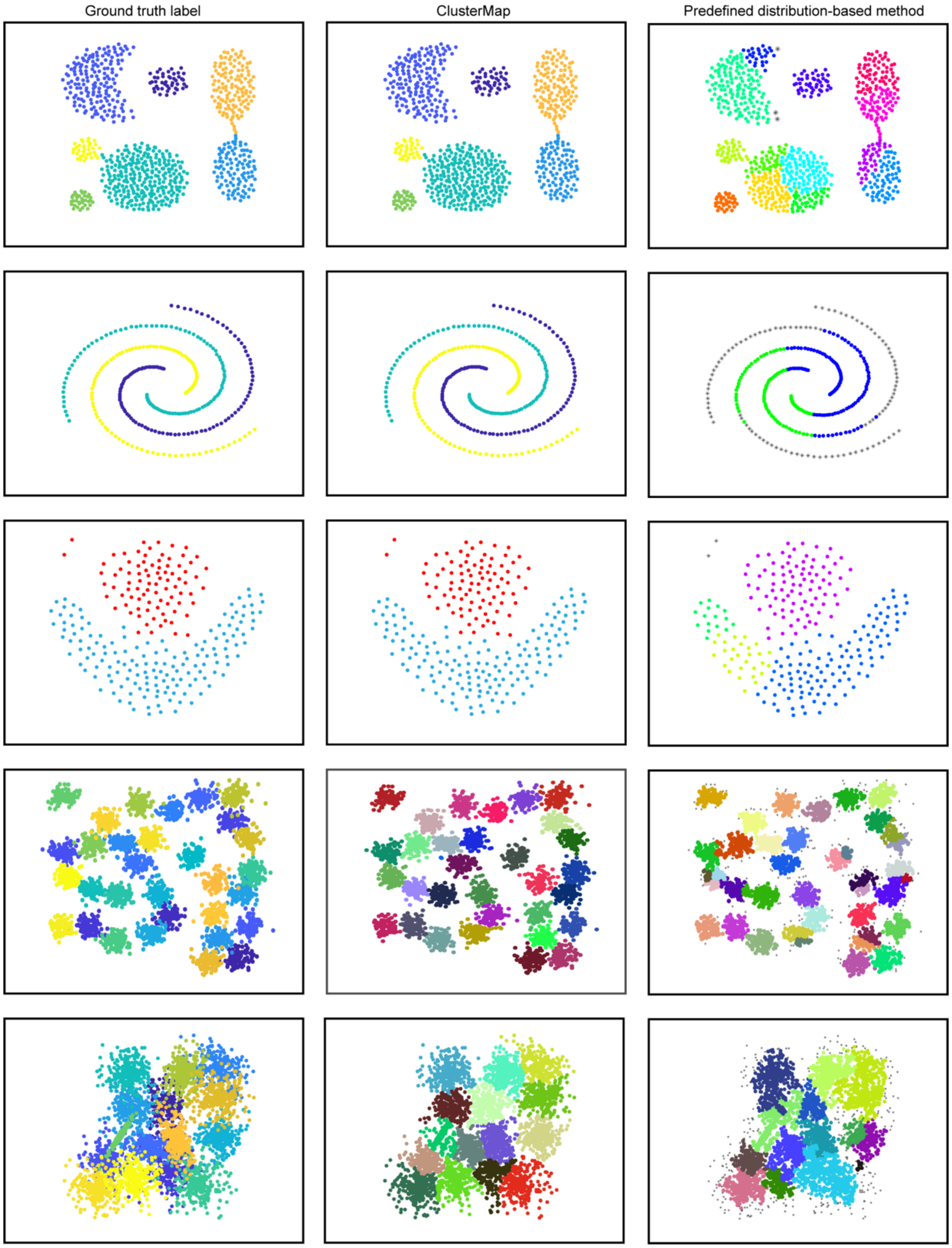
Ground truth label
ClusterMap
Predefined distribution-based method

Supplementary Figure 1

Performance comparison of ClusterMap and predefined distribution-based method in simulated data. Different colors represent different segmentation results. Note that the gene identity of each spot is randomly assigned from 1 to 5 as pseudo gene type. Left: ground truth; Middle: ClusterMap results; Right: results using predefined distribution-based method[20].

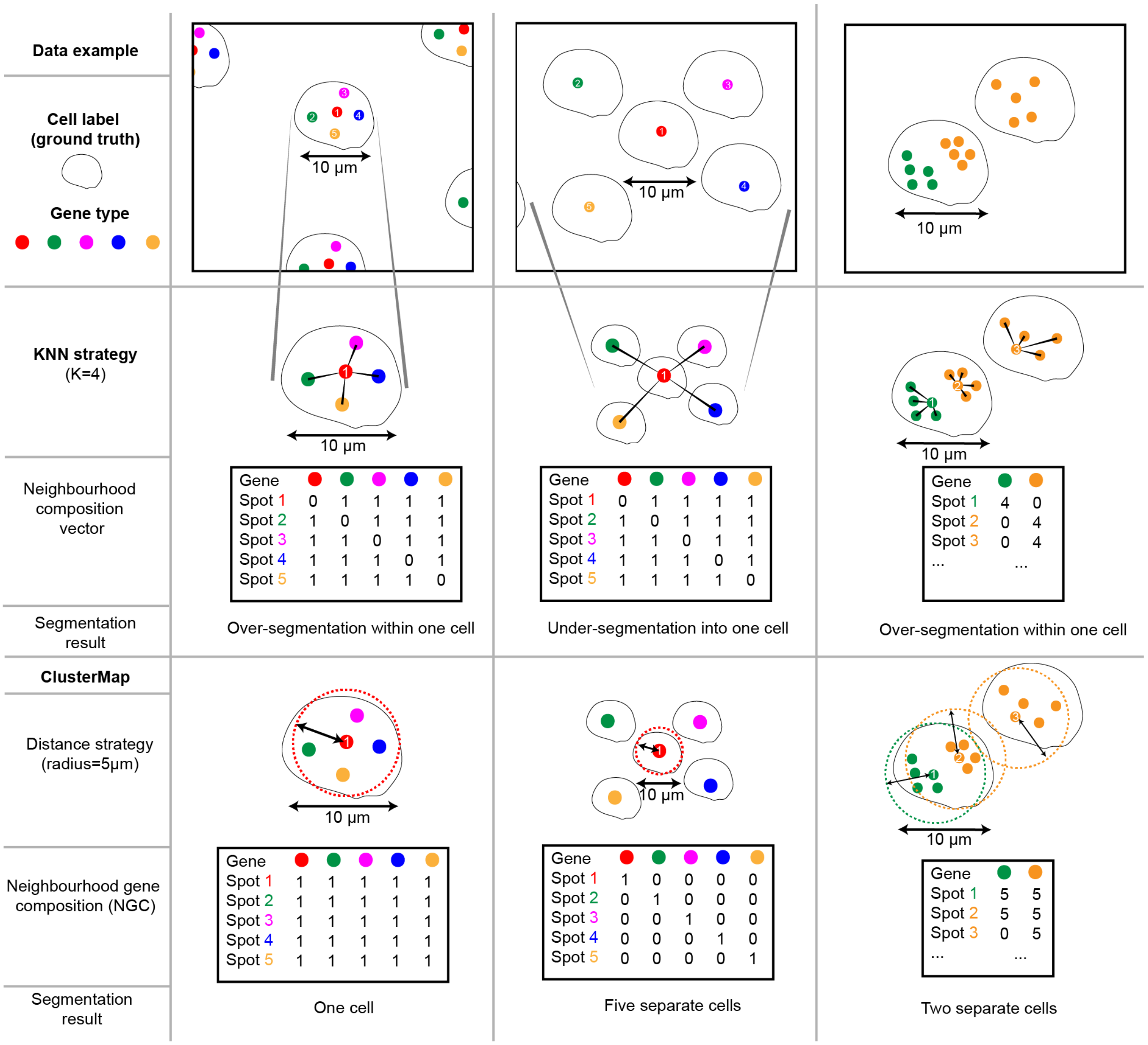
Data example
Cell label (ground truth)
Gene type
10 µm
KNN strategy (K=4)
Neighbourhood composition vector
Gene
Spot 1 0 1 1 1 1
Spot 2 1 0 1 1 1
Spot 3 1 1 0 1 1
Spot 4 1 1 1 0 1
Spot 5 1 1 1 1 0
Gene
Spot 1 0 1 1 1 1
Spot 2 1 0 1 1 1
Spot 3 1 1 0 1 1
Spot 4 1 1 1 0 1
Spot 5 1 1 1 1 0
Gene
Spot 1 4 0
Spot 2 0 4
Spot 3 0 4
... ...
Segmentation result
Over-segmentation within one cell
Under-segmentation into one cell
Over-segmentation within one cell
ClusterMap
Distance strategy (radius=5µm)
Neighbourhood gene composition (NGC)
Gene
Spot 1 1 1 1 1 1
Spot 2 1 1 1 1 1
Spot 3 1 1 1 1 1
Spot 4 1 1 1 1 1
Spot 5 1 1 1 1 1
Gene
Spot 1 1 0 0 0 0
Spot 2 0 1 0 0 0
Spot 3 0 0 1 0 0
Spot 4 0 0 0 1 0
Spot 5 0 0 0 0 1
Gene
Spot 1 5 5
Spot 2 5 5
Spot 3 0 5
... ...
Segmentation result
One cell
Five separate cells
Two separate cells

Supplementary Figure 2

Comparison of RNA sampling approaches between ClusterMap using absolute physical distance with other methods[20] using *k*-nearest neighbours (kNN) in simulated data. Three examples of RNAs with various local density demonstrating that ClusterMap preserves the local physical density information while kNN does not consider the physical density of RNAs.

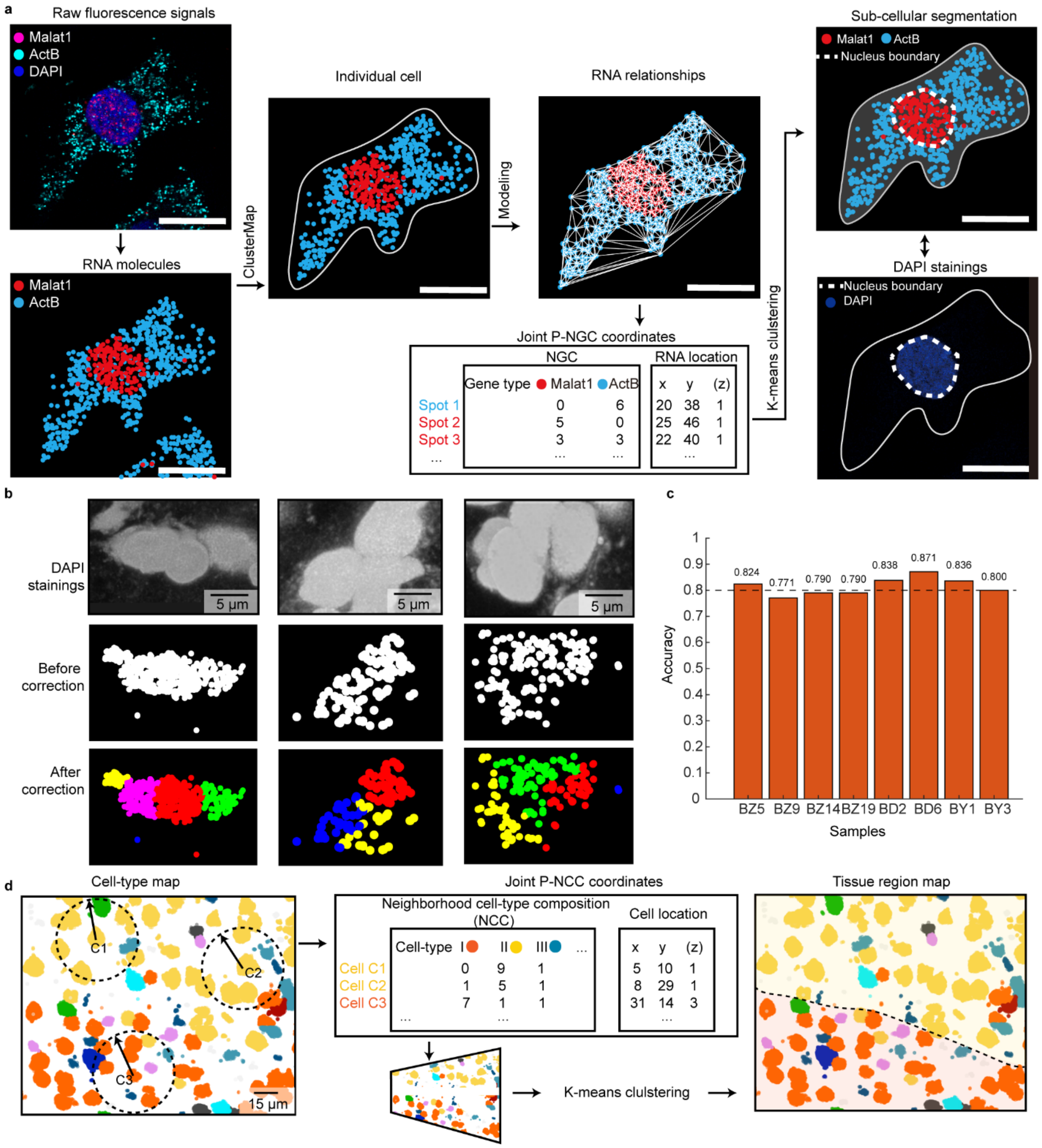
a
Raw fluorescence signals
Malat1
ActB
DAPI
RNA molecules
Malat1
ActB
ClusterMap
Individual cell
Modeling
RNA relationships
Joint P-NGC coordinates
NGC
RNA location
Gene type
Malat1
ActB
x y (z)
Spot 1 0 6 20 38 1
Spot 2 5 0 25 46 1
Spot 3 3 3 22 40 1
...
K-means clustering
Sub-cellular segmentation
Malat1
ActB
Nucleus boundary
DAPI stainings
Nucleus boundary
DAPI
b
DAPI stainings
5 μm
5 μm
5 μm
Before correction
After correction
c
Accuracy
0.824 0.771 0.790 0.790 0.838 0.871 0.836 0.800
BZ5 BZ9 BZ14 BZ19 BD2 BD6 BY1 BY3
Samples
d
Cell-type map
C1
C2
C3
15 μm
Joint P-NCC coordinates
Neighborhood cell-type composition (NCC)
Cell location
Cell-type I II III ...
x y (z)
Cell C1 0 9 1 5 10 1
Cell C2 1 5 1 8 29 1
Cell C3 7 1 1 31 14 3
...
K-means clulstering
Tissue region map

Supplementary Figure 3

Illustration of sub-cellular, cellular, and tissue region analyses. a, Subcellular analysis process of the panel IV in **Figure 1d** by ClusterMap. A three-channel (magenta: *Malat1*; cyan: *ActB*; blue: DAPI) composite image shows raw fluorescent signals. After preprocessing mRNA molecules with specific genes located, ClusterMap first performs cellular resolution and identifies individual cells. Then a mesh graph that models the relationships among mRNA spots in the cell is generated to compute the NGC coordinates and *K*-means clustering separate spots into two regions using joint physical and NGC coordinates. 100 times K-means clustering was performed with different seeds and showed the consistent same results. Finally, a convex hull is constructed from the nucleus spots, denoting the nucleus boundary. The pattern of ClusterMap-constructed nucleus boundary is compared with the DAPI staining. Scale bar: 20μm. **b**, Examples of the cell identification in ClusterMap procedures in **Fig. 2a**. Upper: DAPI staining showing the cell nuclei. Middle: mRNA spots. Lower: Clustering results. **c**, The accuracy of cell identification results from eight STARmap[6] datasets compared with corresponding expert-annotated labels. BZ5, BZ9, BZ14, BZ19: four STARmap[6] 166-gene sets in mouse medial prefrontal cortex (mPFC); BD2, BD6: two STARmap[6] 160-gene sets in mouse V1. BY1, BY3: two STARmap 1020-gene sets in mouse V1. The horizontal line is at 80% accuracy. **d**, ClusterMap constructs the tissue regions after cell-typing. First, the neighborhood cell-type composition (NCC) of each cell is computed by considering a sliding window over the cell-type map. Then both the NCC and physical locations of cells are combined for *K*-means clustering. Cells with highly correlated neighboring cell-type composition and close spatial distances are merged into a single tissue region signature.

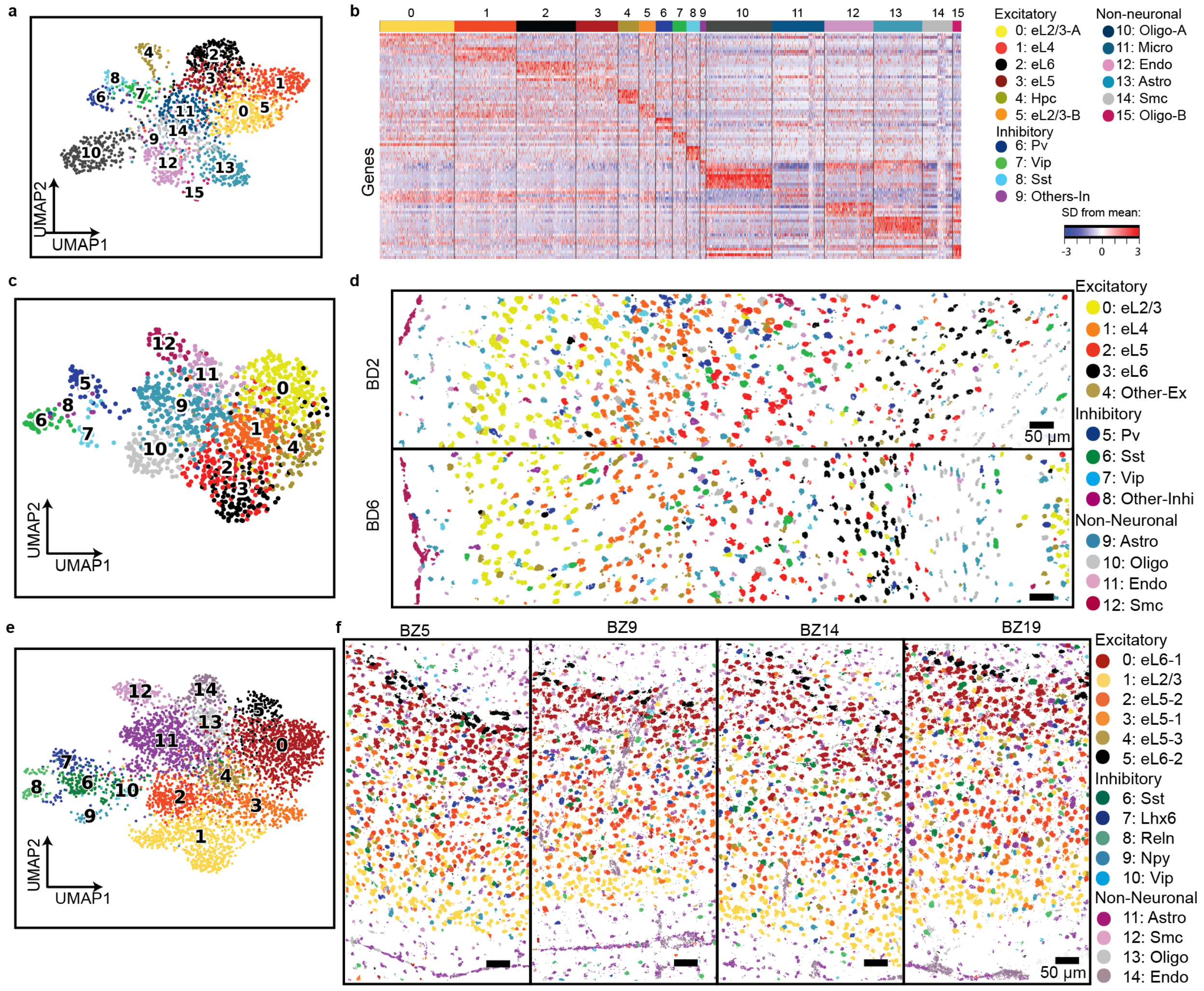
a
b
Genes
UMAP2
UMAP1
Excitatory
0: eL2/3-A
1: eL4
2: eL6
3: eL5
4: Hpc
5: eL2/3-B
Inhibitory
6: Pv
7: Vip
8: Sst
9: Others-In
Non-neuronal
10: Oligo-A
11: Micro
12: Endo
13: Astro
14: Smc
15: Oligo-B
SD from mean:
c
d
BD2
BD6
50 µm
Excitatory
0: eL2/3
1: eL4
2: eL5
3: eL6
4: Other-Ex
Inhibitory
5: Pv
6: Sst
7: Vip
8: Other-Inhi
Non-Neuronal
9: Astro
10: Oligo
11: Endo
12: Smc
e
f
BZ5
BZ9
BZ14
BZ19
50 µm
Excitatory
0: eL6-1
1: eL2/3
2: eL5-2
3: eL5-1
4: eL5-3
5: eL6-2
Inhibitory
6: Sst
7: Lhx6
8: Reln
9: Npy
10: Vip
Non-Neuronal
11: Astro
12: Smc
13: Oligo
14: Endo

Supplementary Figure 4

Identification of cell types in mouse primary cortex (V1) and medial prefrontal cortex (mPFC). a,**b**, UMAP and heatmap visualization of all excitatory, inhibitory and non-neuronal cell types in STARmap[6] mouse V1 1020-gene (two replicates: BY1 and BY3). The heatmap represents z-scored expression matrix of all cell types, showing clustering of five most differentially expressed genes per cell type. Genes shown are selected based on a false discovery rate (FDR)-adjusted p-value threshold of 0.05 (Benjamini-Hochberg correction) and a minimum log_{10} fold change of 0.4, using a two-sided, unpaired t-test, for genes that are expressed in cells within each cluster versus cells in any other cluster. **c**, **e**, UMAP visualization of all excitatory, inhibitory and non-neuronal cell types in STARmap[6] 160-gene datasets in mouse V1 (two replicates: BD2, BD6, (**c**)), and STARmap[6] 166-gene datasets in mPFC (four replicates, BZ5, BZ9, BZ14, BZ19, (**e**)). **d**, **f**, Spatial organization map of cell types in BD2 and BD6 (**d**), and in BZ5, BZ9, BZ14 and BZ19 (**f**).

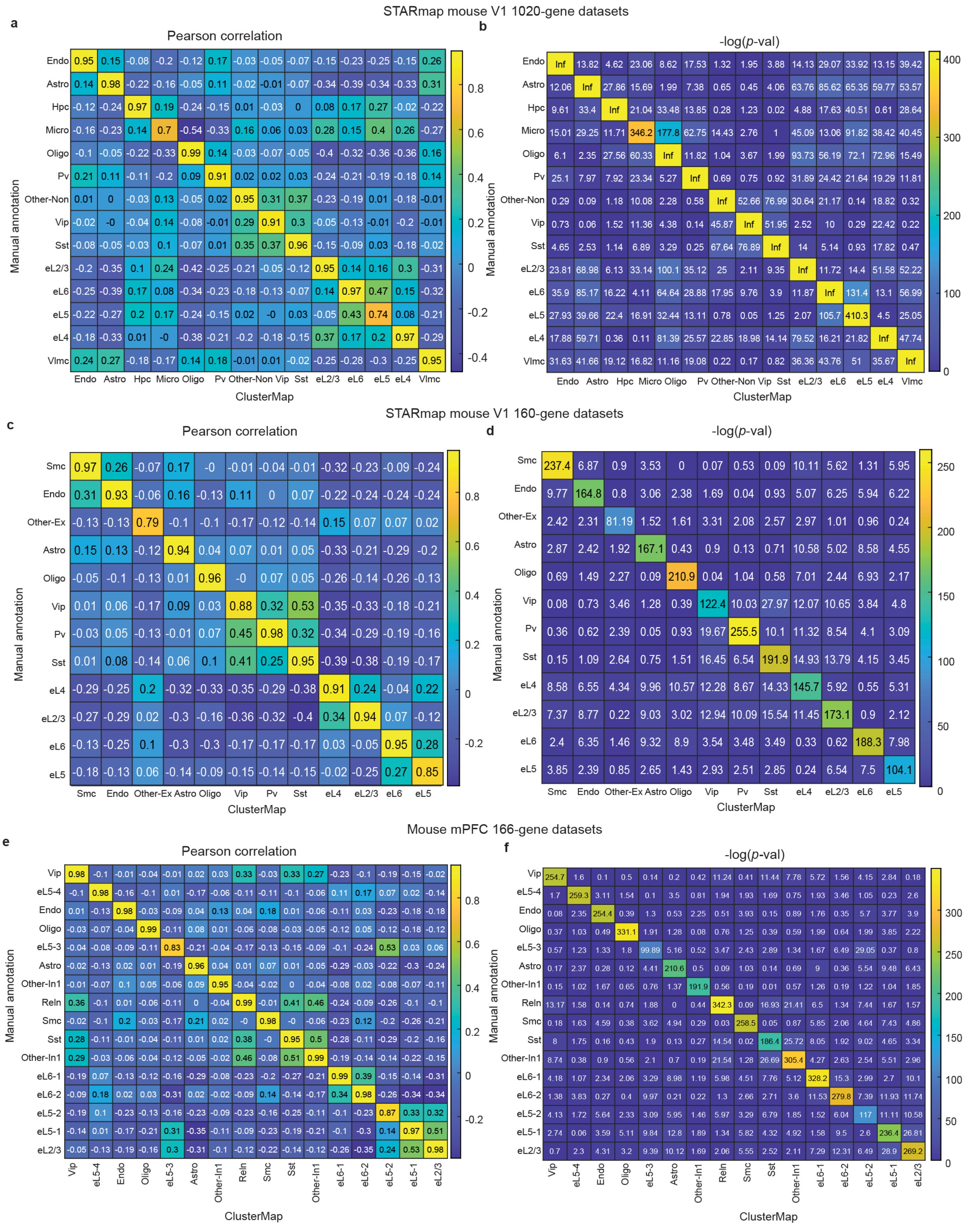

STARmap mouse V1 1020-gene datasets
a
Pearson correlation
b
-log(p-val)
Manual annotation
ClusterMap
Endo Astro Hpc Micro Oligo Pv Other-Non Vip Sst eL2/3 eL6 eL5 eL4 Vlmc
0.8 0.6 0.4 0.2 0 -0.2 -0.4
400 300 200 100 0
STARmap mouse V1 160-gene datasets
c
Pearson correlation
d
-log(p-val)
Smc Endo Other-Ex Astro Oligo Vip Pv Sst eL4 eL2/3 eL6 eL5
0.8 0.6 0.4 0.2 0 -0.2
250 200 150 100 50 0
Mouse mPFC 166-gene datasets
e
Pearson correlation
f
-log(p-val)
Vip eL5-4 Endo Oligo eL5-3 Astro Other-In1 Reln Smc Sst Other-In1 eL6-1 eL6-2 eL5-2 eL5-1 eL2/3
0.8 0.6 0.4 0.2 0 -0.2
300 250 200 150 100 50 0

Supplementary Figure 5

Cell-type correlation matrices comparison of ClusterMap-based and manually-segmented cell types. **a**,**b**, Comparison on STARmap mouse V1 1020-gene datasets. Heatmaps of Pearson correlation (**a**) and -*log*(*p*-value) (**b**) for null hypothesis testing. The *p* value is based on a t statistic which has *n*-2 degrees of freedom and 95% confidence interval. The single-cell gene expression profiles from ClusterMap with manual annotation are compared. **c**,**d**, Comparison on STARmap mouse V1 160-gene datasets. Heatmaps of correlation (**c**) and -*log*(*p*-value) (**d**) comparing the single-cell gene expression profiles from ClusterMap with manual annotation. **e**,**f**, Comparison on STARmap mouse mPFC 166-gene datasets. Heatmaps of correlation (**e**) and -*log*(*p*-value) (**f**) comparing the single-cell gene expression profiles from ClusterMap with manual annotation. Horizontal: ClusterMap; vertical: manual annotation.

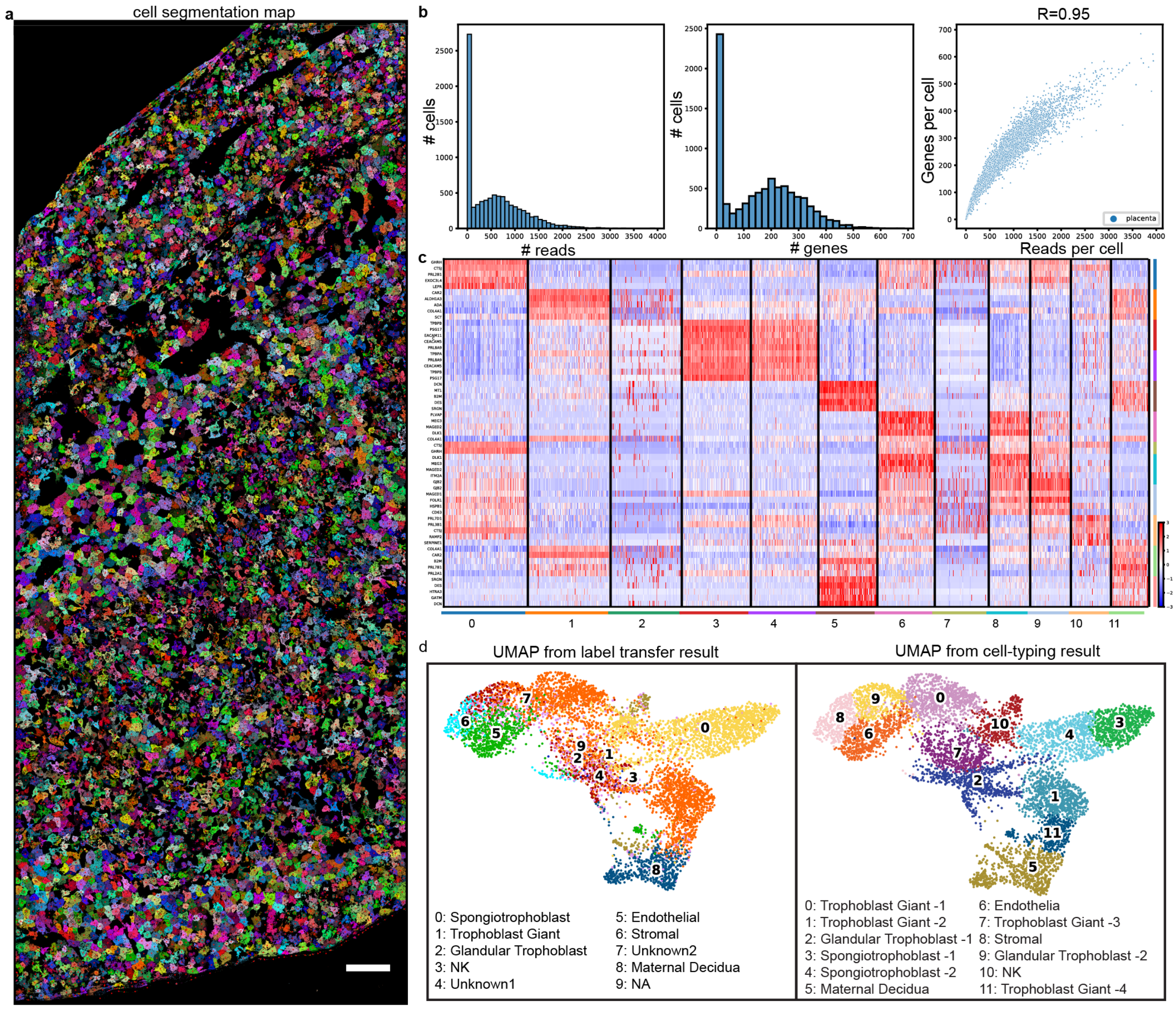
a
cell segmentation map
b
# cells
# reads
# cells
# genes
R=0.95
Genes per cell
Reads per cell
placenta
c
0
1
2
3
4
5
6
7
8
9
10
11
d
UMAP from label transfer result
0: Spongiotrophoblast
1: Trophoblast Giant
2: Glandular Trophoblast
3: NK
4: Unknown1
5: Endothelial
6: Stromal
7: Unknown2
8: Maternal Decidua
9: NA
UMAP from cell-typing result
0: Trophoblast Giant -1
1: Trophoblast Giant -2
2: Glandular Trophoblast -1
3: Spongiotrophoblast -1
4: Spongiotrophoblast -2
5: Maternal Decidua
6: Endothelia
7: Trophoblast Giant -3
8: Stromal
9: Glandular Trophoblast -2
10: NK
11: Trophoblast Giant -4

Supplementary Figure 6

Analyses of the STARmap[6] mouse placental dataset. **a**, ClusterMap generates the cell segmentation map of the STARmap[6] mouse placenta 903-gene dataset, including 7,224 cells. Scale bar: 100 µm. **b**, Statistics of ClusterMap identified placental cells as shown in (**a**). Left: Histogram of detected reads (DNA amplicons) per cell. Middle: Histogram of genes per cell. Right: Correlation plot between genes per cell and reads per cell. **c**, Heatmap visualization of 12 cell types. Names are in the right panel of (**d**). **d**, UMAP from label transfer results with scRNA-seq, compared with UMAP of the Louvain clustering[22] in ClusterMap.

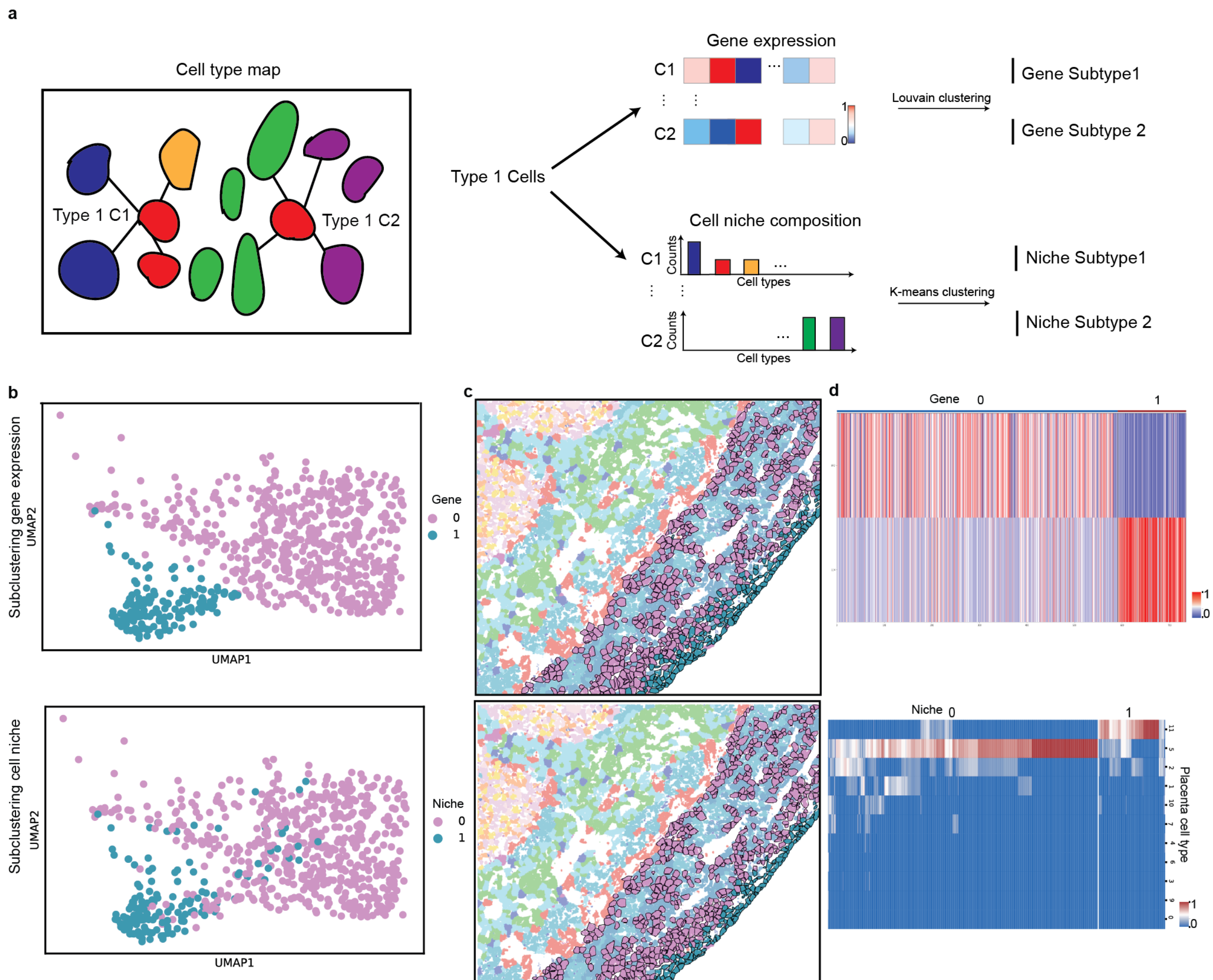
a
Cell type map
Type 1 C1
Type 1 C2
Type 1 Cells
Gene expression
C1
C2
Louvain clustering
Gene Subtype1
Gene Subtype 2
Cell niche composition
Counts
Cell types
K-means clustering
Niche Subtype1
Niche Subtype 2
b
Subclustering gene expression
UMAP2
UMAP1
Gene
0
1
Subclustering cell niche
Niche
c
d
Placenta cell type

93

Supplementary Figure 7

Sub-clustering within one cell type using cell niche compositions in STARmap mouse placenta 903-gene dataset. a, Schematic indicating how cells in one cell type are sub-clustered based on either gene expression (Louvain clustering[22]) or the cell niche compositions (*K*-means clustering[19]). **b**, UMAP of gene expression sub-clustering (top) or cell niche composition sub-clustering (bottom) in Maternal Decidua-1 (MD-1). **c**, Spatial subtype maps using gene expression (top) or cell niche composition (bottom) in MD-1. **d**, Heatmap of sub-clustering using gene expression (top) or cell niche composition sub-clustering (bottom) in MD-1. Gene markers in the top heatmaps of gene expression sub-clustering are 0: *GPNMB*, 1: *CXCL14*. Row names in the bottom heatmaps of cell niche composition sub-clustering are cell types in numbers annotated in Figure 3.

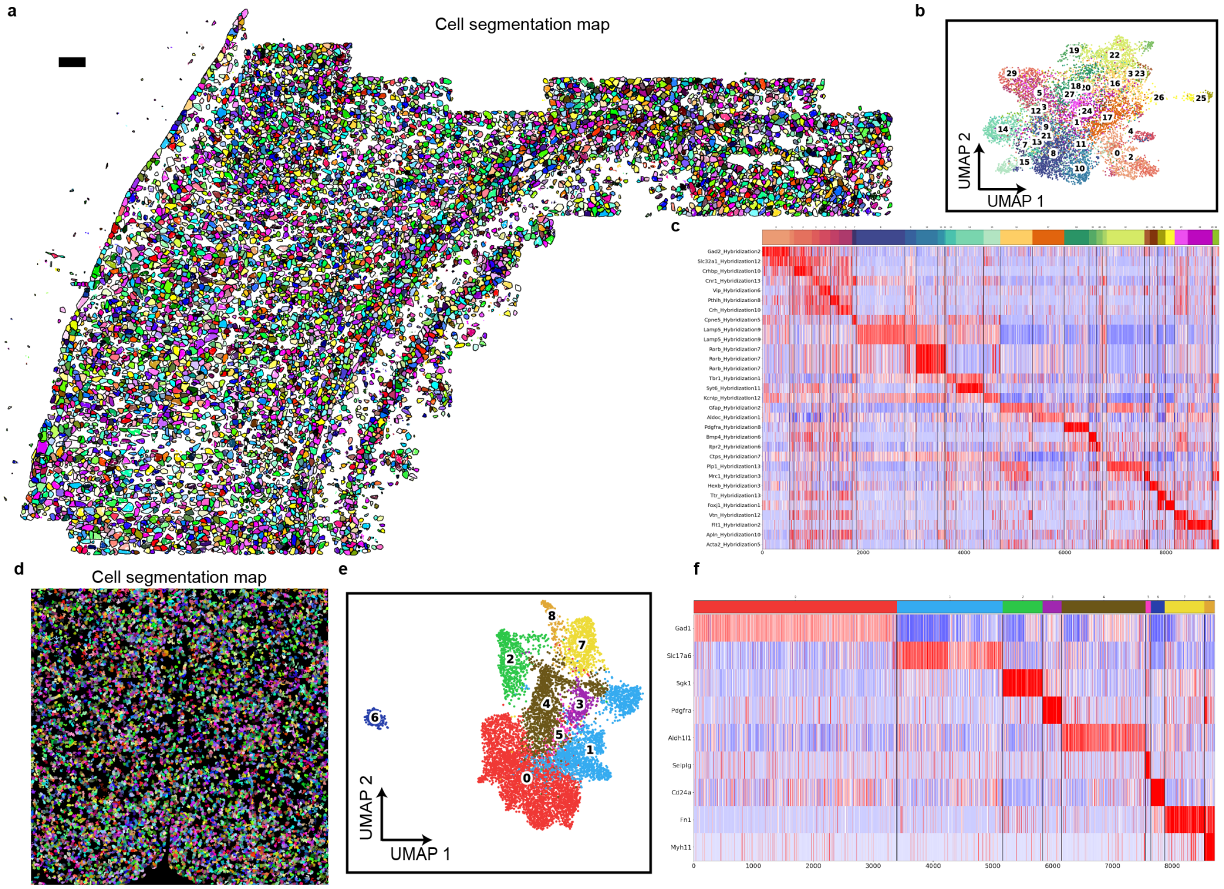
a
Cell segmentation map
b
UMAP 2
UMAP 1
c
Gad2_Hybridization2
Slc32a1_Hybridization12
Crhbp_Hybridization10
Cnr1_Hybridization13
Vip_Hybridization6
Pthlh_Hybridization8
Crh_Hybridization10
Cpne5_Hybridization5
Lamp5_Hybridization9
Lamp5_Hybridization9
Rorb_Hybridization7
Rorb_Hybridization7
Rorb_Hybridization7
Tbr1_Hybridization1
Syt6_Hybridization11
Kcnip_Hybridization12
Gfap_Hybridization2
Aldoc_Hybridization1
Pdgfra_Hybridization8
Bmp4_Hybridization6
Itpr2_Hybridization6
Ctps_Hybridization7
Plp1_Hybridization13
Mrc1_Hybridization3
Hexb_Hybridization3
Ttr_Hybridization13
Foxj1_Hybridization1
Vtn_Hybridization12
Flt1_Hybridization2
Apln_Hybridization10
Acta2_Hybridization5
0
2000
4000
6000
8000
d
Cell segmentation map
e
0
1
2
3
4
5
6
7
8
UMAP 2
UMAP 1
f
Gad1
Slc17a6
Sgk1
Pdgfra
Aldh1l1
Selplg
Cd24a
Fn1
Myh11
0
1000
2000
3000
4000
5000
6000
7000
8000

106

Supplementary Figure 8

ClusterMap analyses across different experimental methods. a, The cell segmentation map of whole osmFISH mouse somatosensory cortex (SSp) datasets. Scale bar: 100 µm. **b,c**, UMAP and heatmap visualization of 31 cell types in osmFISH datasets. **d**, The 2D cell segmentation map of whole MERFISH mouse preoptic area (POA) datasets. Scale bar: 200 µm. **e**,**f**, UMAP and heatmap visualization of 9 cell types in MERFISH datasets. The number of cells increased from 6,471 to 8,538 for osmFISH, from 2,620 to 2,924 for pciSeq, from 6,977 to 10,320 for MERFISH. The number of reads increased from 1,248,106 to 1,690,328 for osmFISH, from 31,246 to 31,750 for pciSeq, from 1,927,913 to 3,065,171 for MERFISH.

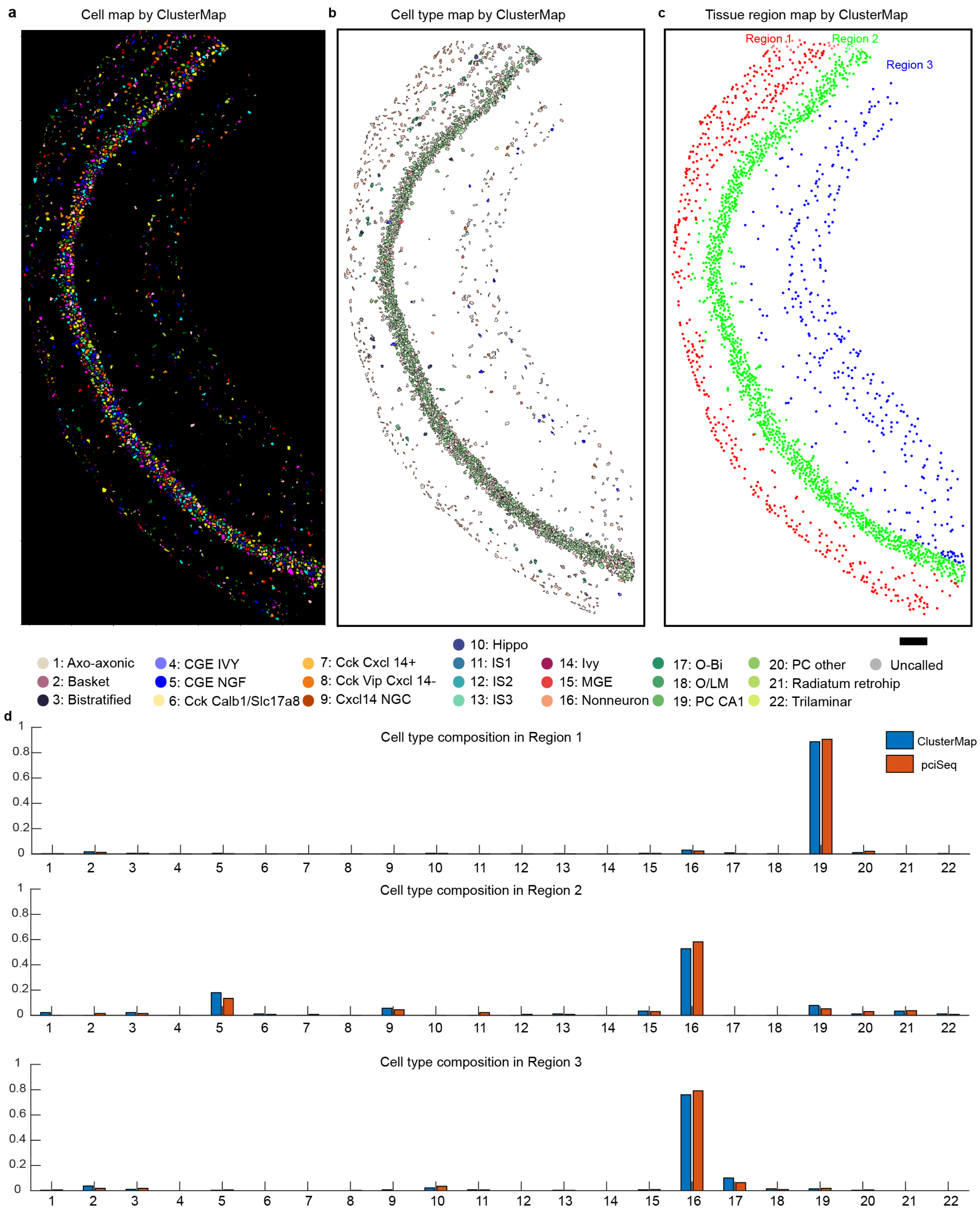

a
Cell map by ClusterMap
b
Cell type map by ClusterMap
c
Tissue region map by ClusterMap
Region 1
Region 2
Region 3
1: Axo-axonic
2: Basket
3: Bistratified
4: CGE IVY
5: CGE NGF
6: Cck Calb1/Slc17a8
7: Cck Cxcl 14+
8: Cck Vip Cxcl 14-
9: Cxcl14 NGC
10: Hippo
11: IS1
12: IS2
13: IS3
14: Ivy
15: MGE
16: Nonneuron
17: O-Bi
18: O/LM
19: PC CA1
20: PC other
21: Radiatum retrohip
22: Trilaminar
Uncalled
d
Cell type composition in Region 1
ClusterMap
pciSeq
Cell type composition in Region 2
Cell type composition in Region 3

Supplementary Figure 9

ClusterMap analyses of ISS data. a, Cell segmentation map shows the cell segmentation results by ClusterMap. Colors are randomly assigned to each cell mask. **b**, Cell type map shows the cell type calling results. Colors are assigned according to their corresponding cell type categories. **c**, Tissue region map shows laminar structure of hippocampus. Scale bar: 200 μm. **d**, Side-by-side comparison of cell type compositions in each tissue region from ClusterMap and pciSeq of the ISS data.

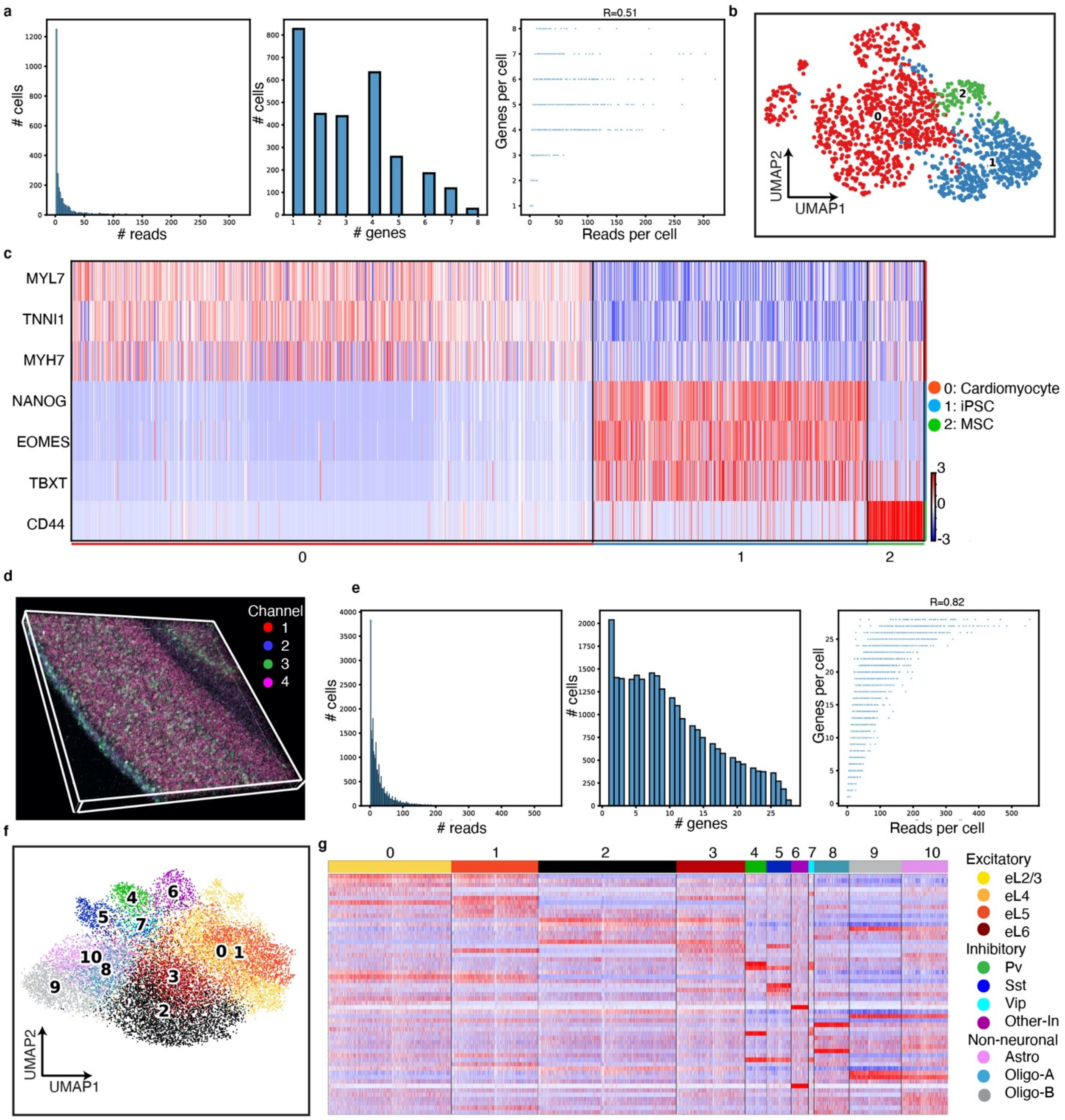

a
# cells
# reads
# cells
# genes
R=0.51
Genes per cell
Reads per cell
b
UMAP2
UMAP1
c
MYL7
TNNI1
MYH7
NANOG
EOMES
TBXT
CD44
0: Cardiomyocyte
1: iPSC
2: MSC
d
Channel
1
2
3
4
e
# cells
# reads
# cells
# genes
R=0.82
Genes per cell
Reads per cell
f
UMAP2
UMAP1
g
Excitatory
eL2/3
eL4
eL5
eL6
Inhibitory
Pv
Sst
Vip
Other-In
Non-neuronal
Astro
Oligo-A
Oligo-B

Supplementary Figure 10

ClusterMap analyses in the 3D datasets. **a**, Statistics of ClusterMap identified cells in the 3D STARmap[6] cardiac organoid[27] 8-gene dataset. Left: Histogram of detected reads (DNA amplicons) per cell. Middle: Histogram of genes per cell. Right: Correlation plot between genes per cell and reads per cell. **b**, **c**, UMAP and heatmap visualization of three cell types in the STARmap[6] cardiac organoid 8-gene dataset. The number of cells in each cell type is as follows: cardiomyocytes, 929; induced pluripotent stem cells (iPSCs), 489; mesenchymal stem cells (MSCs), 101. **d**, 3D four-channel composite raw fluorescent image of the first sequencing round shows spatial arrangement of mRNA molecules in the STARmap[6] mouse V1 28-gene dataset. Width 184 μm, height 194 μm, depth 100 μm. **e**, Statistics of ClusterMap identified cells in (**d**). Left: Histogram of detected reads (DNA amplicons) per cell. Middle: Histogram of genes per cell. Right: Correlation plot between genes per cell and reads per cell. **f**, **g**, UMAP and heatmap visualization of three cell types of (**d**).

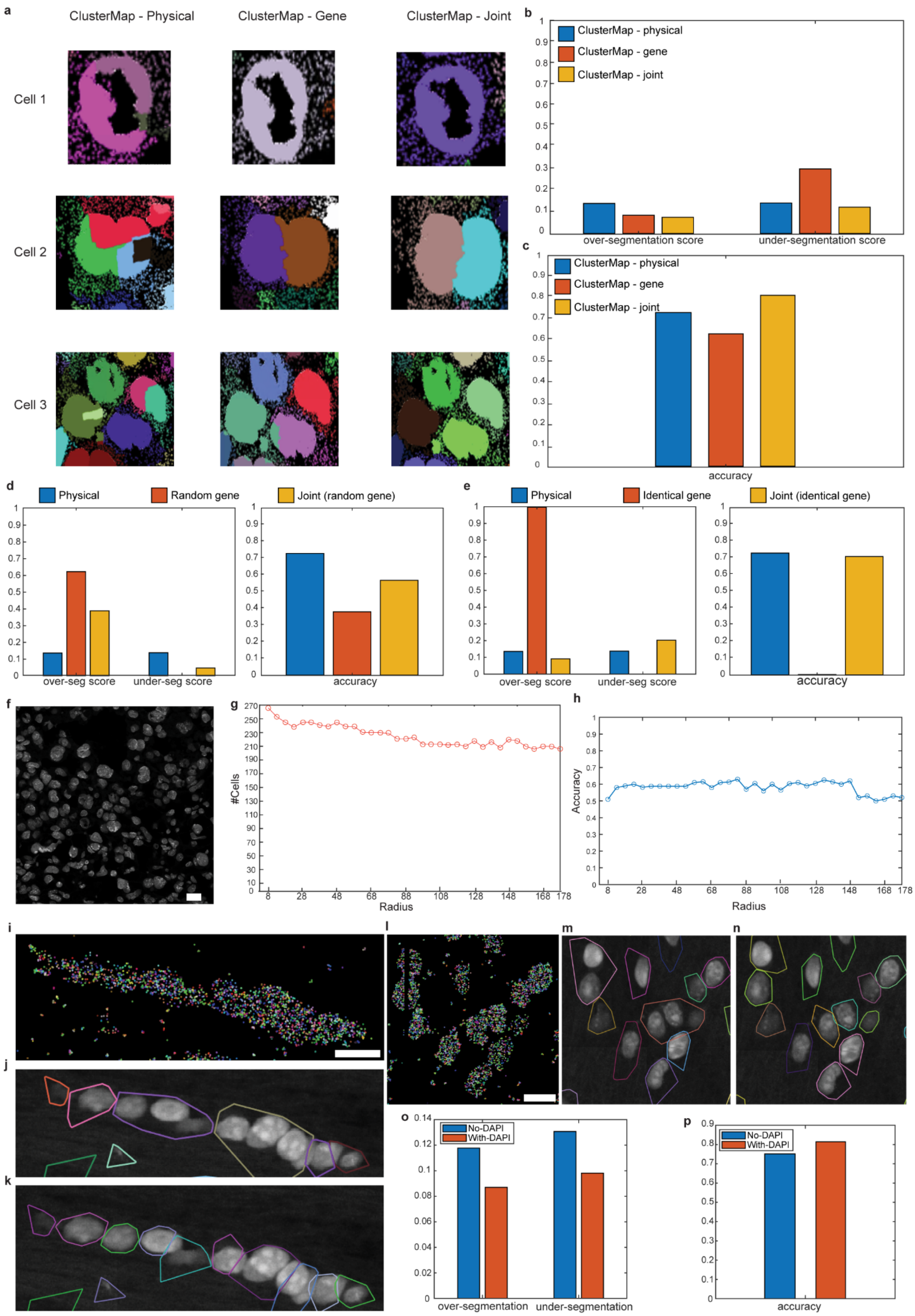
a
ClusterMap - Physical
ClusterMap - Gene
ClusterMap - Joint
Cell 1
Cell 2
Cell 3
b
ClusterMap - physical
ClusterMap - gene
ClusterMap - joint
over-segmentation score
under-segmentation score
c
ClusterMap - physical
ClusterMap - gene
ClusterMap - joint
accuracy
d
Physical
Random gene
Joint (random gene)
over-seg score
under-seg score
accuracy
e
Physical
Identical gene
Joint (identical gene)
over-seg score
under-seg score
accuracy
f
g
#Cells
Radius
h
Accuracy
Radius
i
j
k
l
m
n
o
No-DAPI
With-DAPI
over-segmentation
under-segmentation
p
No-DAPI
With-DAPI
accuracy

Supplementary Figure 11

Performance comparison of ClusterMap using physical density, gene distance, and joint information. a, Examples of cell segmentation using only physical density information (left), gene (NGC) distance information (middle), and joint information (right). **b**,**c**, Bar plots demonstrating the percentage of over-/under- segmented cells in ground truth cells (**b**) and overall accuracy (**c**) in using physical distances information, gene (NGC) distance information, and joint information. **d**,**e**, Bar plots demonstrating the percentage of over-/under- segmented cells in ground truth cells (left) and overall accuracy (right) in using physical distances information, random (**d**) or identical (**e**) gene (NGC) distance information, and joint information. **f**, Raw DAPI image of the targeted mouse placenta tissue. Scale bar: 20μm. **g**,**h**, Line plots showing the number of cells and overall accuracy to the radius. **i-m**, Two examples of the hippocampus regions in STARmap mouse V1 1020-gene datasets showing raw spatial transcriptomics data (**i**,**l**), ClusterMap results without DAPI (**j**,**m**), and ClusterMap results with DAPI (**k**,**n**). Scale bar: 20 μm. **o**,**p**, Bar plots showing the percentage of over-/under- segmented cells (**o**) and overall accuracy (**p**) from ClusterMap without and with DAPI.

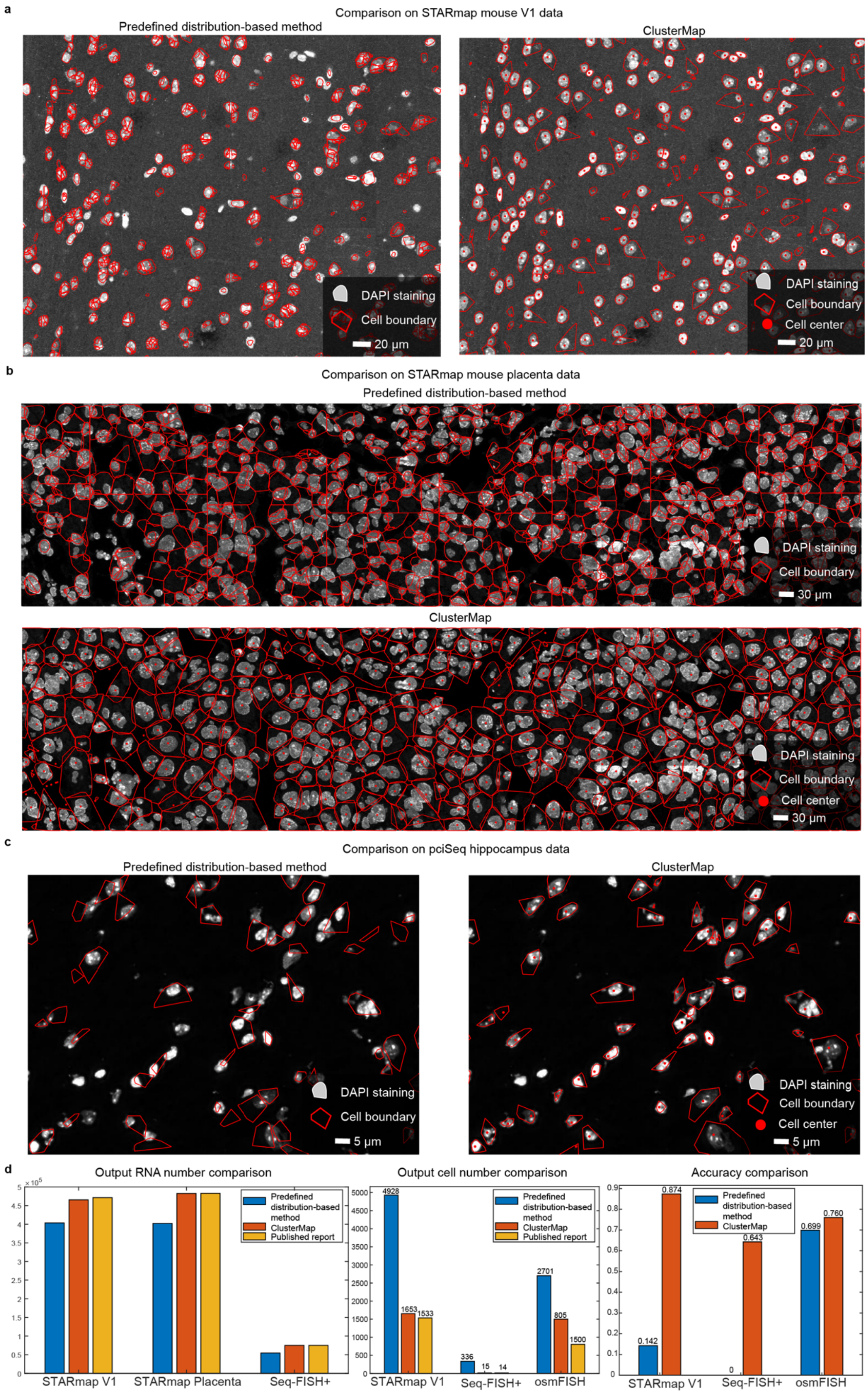
a
Comparison on STARmap mouse V1 data
Predefined distribution-based method
ClusterMap
DAPI staining
Cell boundary
Cell center
20 μm
b
Comparison on STARmap mouse placenta data
Predefined distribution-based method
ClusterMap
30 μm
c
Comparison on pciSeq hippocampus data
5 μm
d
Output RNA number comparison
Output cell number comparison
Accuracy comparison
Predefined distribution-based method
Published report
STARmap V1
STARmap Placenta
Seq-FISH+
osmFISH
4928
1653
1533
336
15
14
2701
805
1500
0.142
0.874
0
0.643
0.699
0.760

Supplementary Figure 12

Performance comparison of ClusterMap and other method across different types of *in situ* transcriptomic data. a, Example of a region in the STARmap[6] mouse V1 1020-gene dataset with DAPI signals (gray) showing ground truth cell nuclei. Red contours show cell boundaries identified by predefined distribution-based method[20] (left) and ClusterMap (right), respectively. **b,c**, As in (**a)** but using the STARmap[6] mouse placenta 903-gene dataset and published pciSeq[4] dataset. **d**, Bar plots demonstrating the remaining RNA numbers, cell numbers and segmentation accuracy for each dataset. In each bar plot, results from predefined distribution-based method[20], ClusterMap, and published reports were shown in blue, red, and yellow, respectively.

Supplementary Table

Supplementary Table 1. Summary of the name, *in situ* sequencing protocol, number of genes, number of cells, number of reads, number of cell types, corresponding figures and note of 7 datasets.

Dataset	Experimental Method	# Genes	# Cells	# Reads	# Cell types	Figures	Note
STARmap mouse V1 1020-gene	STARmap	1,020	1,599	863,426	16	Fig. 1c, Fig. 2, Supplementary Figs. 3,4,5	Source: Ref. 6. 2D analysis
STARmap mouse placenta 903-gene	STARmap	903	7,224	5,090,930	12	Fig. 3, Fig. 4, Supplementary Figs. 6,7	New data. 2D analysis
MERFISH mouse POA	MERFISH	140	10,320	3,065,171	9	Fig. 5, Supplementary Fig. 8	Source: Ref. 3. 3D analysis
pciSeq mouse hippocampus	ISS	98	2,924	31,750	23	Fig. 5, Supplementary Fig. 9	Source: Ref. 4. 2D analysis
osmFISH mouse SSp	osmFISH	33	8,538	1,690,328	31	Fig. 5, Supplementary Fig. 8	Source: Ref. 5. 2D analysis
STARmap cardiac organoid 8-gene	STARmap	8	1,519	47,594	3	Fig. 6, Supplementary Fig. 10	New data, 3D analysis
STARmap mouse V1 28-gene	STARmap	28	24,590	753,396	11	Fig. 6, Supplementary Fig. 10	Source: Ref. 6. 3D analysis
