# Supplementary material for: ClusterMap for multi-scale clustering analysis of spatial gene expression
Source: Nat Commun. 2021 Oct 8;12:5909. doi: 10.1038/s41467-021-26044-x (PMC8501103; doi:10.1038/s41467-021-26044-x)
Supplement: Supplementary file 2 — Reporting Summary [file 41467_2021_26044_MOESM2_ESM.pdf]

nature research

Corresponding author(s): Xiao Wang, Jia Liu

Last updated by author(s): Sep 7, 2021

Reporting Summary

Nature Research wishes to improve the reproducibility of the work that we publish. This form provides structure for consistency and transparency in reporting. For further information on Nature Research policies, see our Editorial Policies and the Editorial Policy Checklist.

Statistics

For all statistical analyses, confirm that the following items are present in the figure legend, table legend, main text, or Methods section.

n/a	Confirmed	
☐	☒	The exact sample size (*n*) for each experimental group/condition, given as a discrete number and unit of measurement
☐	☒	A statement on whether measurements were taken from distinct samples or whether the same sample was measured repeatedly
☐	☒	The statistical test(s) used AND whether they are one- or two-sided<br>*Only common tests should be described solely by name; describe more complex techniques in the Methods section.*
☒	☐	A description of all covariates tested
☐	☒	A description of any assumptions or corrections, such as tests of normality and adjustment for multiple comparisons
☐	☒	A full description of the statistical parameters including central tendency (e.g. means) or other basic estimates (e.g. regression coefficient) AND variation (e.g. standard deviation) or associated estimates of uncertainty (e.g. confidence intervals)
☐	☒	For null hypothesis testing, the test statistic (e.g. *F*, *t*, *r*) with confidence intervals, effect sizes, degrees of freedom and *P* value noted<br>*Give P values as exact values whenever suitable.*
☒	☐	For Bayesian analysis, information on the choice of priors and Markov chain Monte Carlo settings
☒	☐	For hierarchical and complex designs, identification of the appropriate level for tests and full reporting of outcomes
☐	☒	Estimates of effect sizes (e.g. Cohen's *d*, Pearson's *r*), indicating how they were calculated

Our web collection on statistics for biologists contains articles on many of the points above.

Software and code

Policy information about availability of computer code

Data collection	Data was collected and processed using the publicly available STARmap tool based on Wang, X. et al. Three-dimensional intact-tissue sequencing of single-cell transcriptional states. Science 361, eaat 5691 (2018).
Data analysis	ClusterMap is implemented based on MATLAB R2019b and Python 3.6. The following packages and software were used in data analysis: UCSF ChimeraX 1.0, ImageJ 1.51, MATLAB R2019b, R 4.0.4, Rstudio 1.4.1106, , Jupyter Notebook 6.0.3, Anaconda 2-2-.02, h5py 3.1.0, hdbscan 0.8.36, hdf5 1.10.4, matplotlib 3.1.3, seaborn 0.11.0, scanpy 1.6.0, numpy 1.19.4, scipy 1.6.3, pandas 1.2.3, scikit-learn 0.22, umap-learn0.4.3, pip 21.0.1, numba 0.51.2, tifffile 2020.10.1, scikit-image 0.18.1, itertools 8.0.0. Codes are available at Code Ocean https://codeocean.com/capsule/6072400/ [Ref. 46].

For manuscripts utilizing custom algorithms or software that are central to the research but not yet described in published literature, software must be made available to editors and reviewers. We strongly encourage code deposition in a community repository (e.g. GitHub). See the Nature Research guidelines for submitting code & software for further information.

Data

Policy information about availability of data

All manuscripts must include a data availability statement. This statement should provide the following information, where applicable:

- Accession codes, unique identifiers, or web links for publicly available datasets
- A list of figures that have associated raw data
- A description of any restrictions on data availability

The MERFISH mouse POA set, osmFISH mouse SSp set, and pciSeq mouse isocortex set are available as supplementary information file of the original manuscript. MERFISH mouse POA set is at http://zhuang.harvard.edu/merfish.html, osmFISH mouse SSp set is at http://linnarssonlab.org/osmFISH, and pciSeq mouse isocortex set is at https://figshare.com/s/88a0fc8157aca0c6f0e8. The STARmap mouse V1 1020-gene, STARmap mouse V1 28-gene set, STARmap cardiac organoid, and

STARmap mouse placenta datasets are available at Code Ocean [Ref. 46].

Field-specific reporting

Please select the one below that is the best fit for your research. If you are not sure, read the appropriate sections before making your selection.

- [x] Life sciences
- [ ] Behavioural & social sciences
- [ ] Ecological, evolutionary & environmental sciences

For a reference copy of the document with all sections, see nature.com/documents/nr-reporting-summary-flat.pdf

Life sciences study design

All studies must disclose on these points even when the disclosure is negative.

Sample size	ClusterMap was validated across 7 spatial transcriptomics datasets. Here, we demonstrated the tool on 5 previously published high quality datasets including STARmap mouse V1 1020-gene, MERFISH mouse POA, pciSeq mouse hippocampus, osmFISH mouse SSp and STARmap mouse V1 28-gene, and 2 novel datasets including STARmap mouse placenta 903-gene and STARmap cardiac organoid 8-gene. We tested in both two- and three-dimensional space, and on diverse tissue types, including mouse brain, placenta, gut, and human cardiac organoids, where the number of genes in the datasets range from 8 to 1020. We also demonstrated ClusterMap to be applicable to various in situ transcriptomic measurements including STARmap, MERFISH, osmFISH and pciSeq.
Data exclusions	N/A. No data was excluded from the study.
Replication	ClusterMap was validated across 7 spatial transcriptomics datasets.
Randomization	N/A. We didn't have multiple experimental groups across biological samples.
Blinding	N/A. We didn't have multiple experimental groups across biological samples.

Reporting for specific materials, systems and methods

We require information from authors about some types of materials, experimental systems and methods used in many studies. Here, indicate whether each material, system or method listed is relevant to your study. If you are not sure if a list item applies to your research, read the appropriate section before selecting a response.

Materials & experimental systems

n/a	Involved in the study	
x		Antibodies
	x	Eukaryotic cell lines
x		Palaeontology and archaeology
	x	Animals and other organisms
x		Human research participants
x		Clinical data
x		Dual use research of concern

Methods

n/a	Involved in the study	
x		ChIP-seq
x		Flow cytometry
x		MRI-based neuroimaging

Eukaryotic cell lines

Policy information about cell lines

Cell line source(s)	HeLa cells (ATCC, CCL-2, lot 70016358)
Authentication	The cell line has been authenticated by the STR method.
Mycoplasma contamination	The cell lines were confirmed as mycoplasma negative by DAPI (4′,6-diamidino-2-phenylindole) or Hoechst DNA staining and microscope imaging.
Commonly misidentified lines (See ICLAC register)	None.

Animals and other organisms

Policy information about studies involving animals; ARRIVE guidelines recommended for reporting animal research

Laboratory animals	C57BL/6 (female, 8-12 weeks) mice were purchased from the Jackson Laboratory (JAX). Animals were housed 2-5 per cage and kept on a reversed 12 h light-dark cycle with ad libitum food and water. For the mouse placenta dataset, we used snap-frozen tissue sections from C57BL/6J x CAST/EiJ matings and performed STARmap to measure expression of 903 genes on the E14.5 mouse

	placenta tissue slices. Sex: female. Age: E14.5. Strain: C57BL/6J x CAST/EiJ matings. Housing conditions: Mice were housed under standard barrier conditions at the Whitehead Institute for Biomedical Research.
Wild animals	None
Field-collected samples	None
Ethics oversight	All procedures involving animals at the Broad Institute were conducted in accordance with the US National Institute of Health Guide for the Care and Use of Laboratory Animals under protocol number 0255-08-19. Experimental procedures were approved by the Institutional Animal Care and Use Committee of the Broad Institute of MIT and Harvard under protocol number 0255-08-19.

Note that full information on the approval of the study protocol must also be provided in the manuscript.
